# Correcting for Phylogenetic Autocorrelation in Species Sensitivity Distributions

**DOI:** 10.1002/ieam.4207

**Published:** 2019-11-18

**Authors:** Dwayne RJ Moore, Colleen D Priest, Nika Galic, Richard A Brain, Sara I Rodney

**Affiliations:** ^1^ Intrinsik Ltd New Gloucester Maine USA; ^2^ Syngenta Crop Protection Greensboro North Carolina USA; ^3^ Intrinsik Corp Ottawa Ontario Canada

**Keywords:** Species sensitivity distribution, Phylogenetic autocorrelation, Chlorpyrifos, Atrazine

## Abstract

A species sensitivity distribution (SSD) is a cumulative distribution function of toxicity endpoints for a receptor group. A key assumption when deriving an SSD is that the toxicity data points are independent and identically distributed (iid). This assumption is tenuous, however, because closely related species are more likely to have similar sensitivities than are distantly related species. When the response of 1 species can be partially predicted by the response of another species, there is a dependency or autocorrelation in the data set. To date, phylogenetic relationships and the resulting dependencies in input data sets have been ignored in deriving SSDs. In this paper, we explore the importance of the phylogenetic signal in deriving SSDs using a case studies approach. The case studies involved toxicity data sets for aquatic autotrophs exposed to atrazine and aquatic and avian species exposed to chlorpyrifos. Full and partial data sets were included to explore the influences of differing phylogenetic signal strength and sample size. The phylogenetic signal was significant for some toxicity data sets (i.e., most chlorpyrifos data sets) but not for others (i.e., the atrazine data sets, the chlorpyrifos data sets for all insects, crustaceans, and birds). When a significant phylogenetic signal did occur, effective sample size was reduced. The reduction was large when the signal was strong. In spite of the reduced effective sample sizes, significant phylogenetic signals had little impact on fitted SSDs, even in the tails (e.g., hazardous concentration for 5^th^ percentile species [HC5]). The lack of a phylogenetic signal impact occurred even when we artificially reduced original sample size and increased strength of the phylogenetic signal. We conclude that it is good statistical practice to account for the phylogenetic signal when deriving SSDs because most toxicity data sets do not meet the independence assumption. That said, SSDs and HC5s are robust to deviations from the independence assumption. *Integr Environ Assess Manag* 2019;00:1–13. © 2019 The Authors. *Integrated Environmental Assessment and Management* published by Wiley Periodicals, Inc. on behalf of Society of Environmental Toxicology & Chemistry (SETAC)

## KEY POINTS

- The assumption when deriving an SSD that toxicity data points are independent is tenuous because species sensitivity is correlated with taxonomic relatedness.
- We explored the importance of the phylogenetic signal in deriving SSDs using toxicity datasets for aquatic autotrophs exposed to atrazine and aquatic and avian species exposed to chlorpyrifos.
- A significant phylogenetic signal was observed in several of our chlorpyrifos toxicity datasets though not in our atrazine toxicity datasets.
- In spite of the reduced effective sample sizes, significant phylogenetic signals had little impact on fitted distributions (e.g., little change to HC5 values), indicating that SSDs are robust to deviations from the independence assumption.

## INTRODUCTION

A species sensitivity distribution (SSD) is a modeled cumulative distribution function of toxicity endpoints for a specified receptor group. In practice, the endpoints (e.g., LC50s) are assumed to have negligible uncertainty and are treated as fixed values. Species sensitivity distributions are often used in risk assessment to characterize risk to hypothetical sensitive species (e.g., hazardous concentration for 5^th^ percentile species [HC5]) or to entire taxonomic groups or communities.

A generalized linear regression approach has often been used by regulators and others to derive SSDs (e.g., Stephan et al. 1985; Solomon et al. 1996; Giesy et al. 1999; Hall et al. 2000; CCME 2007; Mebane 2010). Although this approach tends to provide conservative estimates of lower tail HC*x* values, several shortcomings have been identified in recent statistical reviews (e.g., Hickey and Craig 2012; Fox 2015, 2016). For example, the approach assumes that the plotting positions of the empirical distribution function are independent observations, when they are in fact a prescribed series of proportions (e.g., Hazen plotting positions). Thus, SSDs do not include a dependent variable and an independent variable as is typically the case with most simple regression analyses. A better approach is to treat the set of toxicity endpoints as a random sample of a single variable, rather than a 2‐variable regression analysis, when deriving an SSD. Estimates of the probability density function for an SSD (e.g., cumulative lognormal distribution) may be derived via maximum likelihood estimation (MLE) (Aldenberg and Slob 1993; Aldenberg and Jaworska 2000). This is the approach we use herein.

Another issue with deriving an SSD from a set of toxicity endpoints is that the data set may not be independent and identically distributed (iid). When fitting distributions, independence of data points is assumed. This assumption is tenuous, however, because closely related species often have similar sensitivities, whereas more distantly related species often have divergent sensitivities (Guenard et al. 2014; Malaj et al. 2016; Esteves et al. 2017; Hylton et al. 2018). This is not surprising given that closely related species are more likely to have shared physiological processes (e.g., detoxification pathways) than do distantly related species (Hylton et al. 2018). When species have a shared evolutionary history, there is a dependency or autocorrelation in the data set. To date, phylogenetic relationships and the resulting dependencies in input data sets have been ignored in deriving SSDs. In analyses involving interspecies comparison of other physiological processes (e.g., metabolic rate vs body weight), ignoring quantitative estimates of phylogenetic signal has been shown to yield statistically significant erroneous parameter estimates in 30% to 50% of cases (e.g., Capellini et al. 2010).

In the present paper, we explore the importance of the phylogenetic signal in deriving SSDs using a case studies approach. The case studies involve toxicity data sets previously assembled for aquatic autotrophic species exposed to atrazine (USEPA 2012; also see Moore et al. 2017) and aquatic and avian species exposed to chlorpyrifos (Giddings et al. 2014; Moore et al. 2014). Full data sets (e.g., all aquatic species in the chlorpyrifos toxicity data set) and partial data sets (e.g., fish only) are included in the analyses to enable exploration of the influences of differing phylogenetic signal strength and sample size. We also include 2 hypothetical data sets to explore whether the influence of a strong phylogenetic signal is greater with very small sample sizes.

The toxicity data sets, the data sets describing the phylogenetic relationships between species in the toxicity data sets, and an example of the R code (R Core Team 2013) used for the statistical analyses are included as Supplemental Data.

## METHODS

### Toxicity data sets

Toxicity data were compiled for aquatic autotrophs exposed to atrazine and for all aquatic and avian receptors exposed to chlorpyrifos.

The aquatic autotrophic species data set for atrazine was previously assembled by the United States Environmental Protection Agency (USEPA 2012). Specific growth rate (SGR)–based EC50s for vascular and nonvascular aquatic autotrophs were used because SGR is independent of exposure time. Although the USEPA (2012) developed the data set for use in a genus sensitivity distribution, information provided in the report was used to match EC50s to species. When testing was not species specific, the genus name was used (e.g., *Najas* sp., *Chlorella* sp., *Cyclotella* sp.). In cases where multiple values were available for a single species, the geometric mean was used. The aquatic autotrophic species data set for atrazine included 33 species from several taxonomic groups, including Angiospermae, Archaeplastida, Chromalveolata, and Cyanobacteria (Supplemental Data Table SI‐1). The SGR EC50s for atrazine ranged from 15 to 706 µg/L. In addition to the aquatic autotrophic species data set, the Angiospermae (*n* = 9) and Arachaeplastida (*n* = 14) data sets were of sufficient size to enable determinations of the influence of the phylogenetic signal on SSD derivation.

Giddings et al. (2014) compiled a data set for aquatic biota exposed to chlorpyrifos. Only studies deemed acceptable for risk assessment were included. The resulting data set had 4 algal, 3 amphibian, 21 crustacean, 25 fish, 16 insect, 3 mollusk, and 1 rotifer species. Effects endpoints (EC50s) ranged from 0.035 to 5174 µg/L. The full data set is provided in Supplemental Data Table SI‐2. In addition to the aquatic species data set, the following data sets were included in further SSD analyses for chlorpyrifos: all vertebrates, all invertebrates, fish, crustaceans, and insects.

Moore et al. (2014) updated the data set previously compiled by Solomon et al. (2001) for acute toxicity of chlorpyrifos to birds. Median lethal doses (LD50s) were compiled from acceptable studies, resulting in endpoints for 14 bird species. The data set included passerines (*n* = 4), gallinaceous species (*n* = 6), waterfowl (*n* = 2), common pigeon (*Columba livia*), and sandhill crane (*Grus canadensis*). Oral LD50s ranged from 5.62 to 122 mg/kg body weight (bw). When multiple LD50s were available for the same species, a geometric mean was used in our analyses. Input values are provided in Supplemental Data Table SI‐3.

### Phylogeny

To determine phylogenetic relationships, the taxonomic identifier (ID) was obtained for each species in the toxicity data sets. This information was obtained from the National Center for Biotechnology Information (NCBI) Taxonomy Browser (NCBI 2019). In some cases, species names had been updated since the completion of the toxicity studies. Current species names and taxonomic IDs were used in our analyses. Genus names and IDs were used when the species names were unknown.

The taxonomic IDs were input to the phyloT generator (Ivica Letunica 2019) to determine and express the phylogenetic relationships among species. The phyloT generator uses taxonomic IDs to access genetic information in the NCBI database. The output is a phylogenetic tree in both picture (.png) and Newick text formats. The phylogenetic trees for aquatic autotrophic species included in the full atrazine data set and for aquatic receptors and birds included in the full chlorpyrifos data sets are shown in Supplemental Data Figures SI‐1 to SI‐3.

### Check for phylogenetic signal

The Newick text files for the phylogenetic relationships determined with phyloT were used to generate a matrix of phylogenetic distances for each species combination. To do this, we used the cophenetic function in the “ape” package in R (Swenson 2014). The resulting matrix has the species names as the row and column names, and the values in the matrix are the sum of the branch lengths separating each pair of species. Thus, if 2 species are far apart with regard to their phylogeny, their distance will be larger and the correlation weaker than that for 2 closely related species.

We next used Moran's *I* index to determine the strength of the phylogenetic signal in each toxicity data set using the BaseTreeStats function in the phylosignal package in R (Keck et al. 2016). The Moran's *I* index is a measure of autocorrelation often used in spatial statistics. The index was proposed as a method for determining the phylogenetic signal by Gittleman and Kot (1990). PhyloSignal computes *I* using Equation 1:

(1) $$I=\frac{n}{\sum_{i=1\;}^{n}\sum_{j=1\;}^{n}w_{\mathrm{ij}}}\frac{\sum_{i=1\;}^{n}\sum_{j=1}^{n}w_{\mathrm{ij}}(y_{i}-\overset{®}{y})(y_{j}-\overset{®}{y})}{{\sum_{i=1}^{n}\left(y_{i}-\overset{®}{y}\right)}^{2}},$$

where *y*_*i*_ and *y*_*j*_ are the trait values (in this case, logged toxicity values) measured for species *i* and species *j*, respectively; *n* is the number of species; and *w*_*ij*_  = 1/*d*_*ij*_, where *d*_*ij*_ is the patristic distance between species *i* and species *j*. The Moran's *I* ranges from 1 to –1. Large negative values are less likely because that would mean closely related species have greater differences in sensitivity to a pesticide than do more distantly related species.

### Effective sample size estimate based on phylogenetic autocorrelation

Assuming the population of logged toxicity endpoints is normally distributed and the sample of logged endpoints being considered is iid, the sample mean and squared standard deviation are the maximum likelihood estimators of the mean and variance of the normal distribution that describes the population (Krishnamoorthy 2015). A significant phylogenetic signal indicates autocorrelation and thus nonindependent samples (i.e., sensitivity is not randomly distributed among taxonomies). In this case, the mean remains an unbiased estimator of the expected value and is asymptotically the best linear unbiased estimator. However, the variance estimate must be adjusted to account for the autocorrelation in the data. This generally leads to a reduction in the effective number of observations, which can be estimated by accounting for the autocorrelation in the data (Zięba and Ramza 2011). The use of an autocorrelation index to correct sample size for a phylogenetic signal is analogous to how samples size is corrected to account for temporal autocorrelation in a monitoring dataset.

If the autocorrelation is first‐order autoregressive, that is the degree of positive autocorrelation between logged endpoints decreases exponentially with distance between species on the phylogenetic tree, then the effective sample size can be estimated as specified by Cressie (1993):

(2) $$n_{\text{eff}}=\frac{n}{1\;+2\left(\frac{\rho }{1-\rho }\right)\left(1-\left(\frac{1}{n}\right)\right)-2{\left(\frac{\rho }{1-\rho }\right)}^{2}\left(\frac{1-\rho^{n-1}}{n}\right)},$$

where, *n*_eff_ = effective sample size (i.e., equivalent number of independent observations); *n* = original sample size (in this case, the number of species toxicity endpoints); and ρ = first‐order autocorrelation parameter.

The autocorrelation parameter ρ can be estimated with Moran's *I* (Münkemüller et al. 2012). A Moran's *I* greater than zero (i.e., closely related species are more similar in sensitivity to a toxicant than are more distantly related species) decreases the effective sample size relative to the number of species in the data set. A Moran's *I* less than zero increases the effective sample size. To our knowledge, the properties of the effective sample size estimator (e.g., bias properties) have not been fully evaluated and thus may warrant further investigation.

### Estimating lognormal SSD parameters with correction for autocorrelation

With the estimate of corrected sample size and assuming a lognormal distribution of endpoints, we can estimate the variance using the following equation (Zięba and Ramza 2011):

(3) $$s_{a}^{2}=\frac{n_{\text{eff}}}{n(n_{e\text{ff}}-1)}\sum_{i=1}^{n}{\left(x_{i}-\overset{®}{x}\right)}^{2},$$

where $s_{a}^{2}$ = estimated variance with correction for effective sample size; $x_{i}$ = logged endpoint of the *i* ^th^ species in the data set; and $\overset{®}{x}$ = mean of logged endpoints in the data set.

Because the mean of the logged endpoints remains an appropriate estimate for the location parameter of the lognormal distribution, we estimated the SSD using a lognormal distribution with location and scale parameters of $\overset{®}{x}$ and $s_{a}^{2}$, respectively.

As is the case with nonautocorrelated data, the lower‐tail estimators (e.g., HC5) from the lognormal and logistic distributions corrected for effective sample size are biased and tend to overestimate the HC5. Correction factors can be obtained from Aldenberg and Slob (1993) and Aldenberg and Jaworska (2000).

## RESULTS

### Atrazine SSDs for aquatic autotrophic species

**Figure 1:**
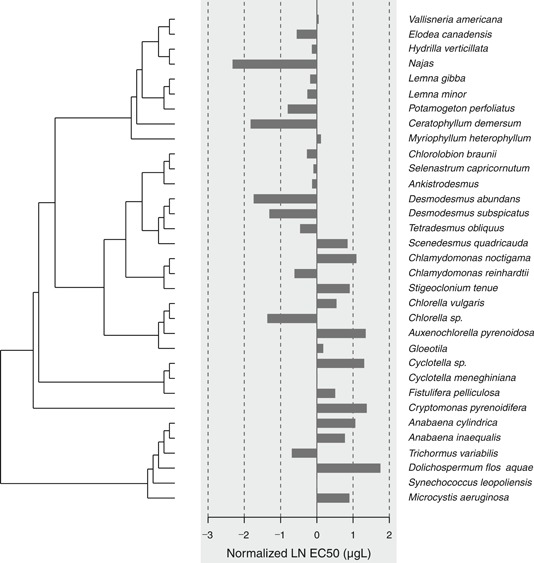
Bar plot showing normalized (i.e., median natural logarithm [LN] EC50 = 0) LN EC50 with respect to the phylogenetic tree for the atrazine all aquatic autotrophic species data set. LN = natural logarithm.

**Figure 2:**
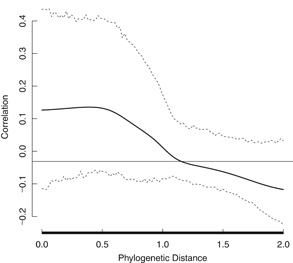
Correlogram for the atrazine all aquatic autotrophic species data set. On the *x*‐axis, red indicates a positive phylogenetic autocorrelation, blue indicates a negative phylogenetic autocorrelation, and black indicates no phylogenetic autocorrelation.

**Figure 3:**
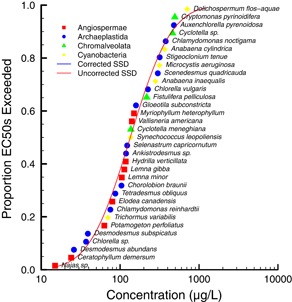
Species sensitivity distribution (SSD) for the atrazine all aquatic autotrophic species data set showing the original, uncorrected SSD and the SSD corrected for phylogenetic autocorrelation.

The influence of phylogenetic autocorrelation was evaluated for a data set of specific growth rate EC50s for 33 species of aquatic autotrophs. The data set included effects endpoints for Angiospermae, Archaeplastida, Chromalveolata, and Cyanobacteria. The phylogenetic tree for the aquatic autotrophs data set with respect to normalized (i.e., median = 0) natural logarithm (LN) EC50 is shown in Figure 1. The correlogram in Figure 2 indicates that there is no phylogenetic autocorrelation for any portion of the data set. As a result, the overall phylogenetic signal, as determined with the Moran's *I* index, was not significant (*p* = 0.077). The effective sample size was 29. The standard deviation of the original data set (0.944) and the data set corrected for effective sample size (0.946) were virtually the same. The HC5 and HC50 of the original (28.5 and 135 µg/L, respectively) and corrected (28.4 and 135 µg/L, respectively) SSDs were also virtually identical (Table 1). The original SSD and SSD corrected for phylogenetic signal are shown in Figure 3 (the SSDs overlap and thus are difficult to distinguish).

**Table 1:** Statistical results for atrazine and chlorpyrifos data sets

Parameter	Atrazine			Chlorpyrifos						
	All aquatic autotrophs	Angiospermae	Archaeplastida	All aquatic species	All vertebrates	All invertebrates	Fish	Insects	Crustaceans	Birds
Sample size	33	9	14	73	28	41	25	16	21	14
Moran's *I*	0.0653	–0.214	–0.111	0.318	0.183	0.293	0.129	0.112	0.0129	0.0499
Moran's *I* (*p*‐value)	0.0770	0.767	0.551	0.001	0.009	0.001	0.0280	0.0630	0.180	0.178
Effective sample size	29	14	17	38	20	23	19	13	20	13
Original standard deviation	0.944	0.812	0.912	3.10	2.35	3.03	2.19	2.21	2.34	0.774
Corrected standard deviation	0.946	0.795	0.905	3.12	2.37	3.06	2.20	2.23	2.34	0.777
HC5 (µg/L)	28.5	19.0	27.9	0.0491	0.693	0.0150	0.646	0.0680	0.0132	8.55
HC50 (µg/L)	135	72.2	125	8.03	33.0	2.18	23.5	2.59	0.622	30.5
Corrected HC5 (µg/L)	28.4	19.5	28.2	0.0475	0.672	0.0142	0.633	0.0661	0.0132	8.51
Corrected HC50 (µg/L)	135	72.2	125	8.03	33.0	2.18	23.5	2.59	0.622	30.5

HC = hazard concentration.

The aquatic autotrophs data set was subdivided to evaluate Angiospermae (Moran's *I*, *p* = 0.767) and Archaeplastida (Moran's *I*, *p* = 0.551) species. Neither subdata set had a significant phylogenetic signal (Table 1), as confirmed by the bar plots and correlograms (see Supplemental Data Figures SI‐4, SI‐5, SI‐7, and SI‐9). The uncorrected and corrected SSDs are shown in Supplemental Data Figures SI‐6 and SI‐9. The statistical results are summarized in Table 1.

### Chlorpyrifos SSDs for aquatic species

**Figure 4:**
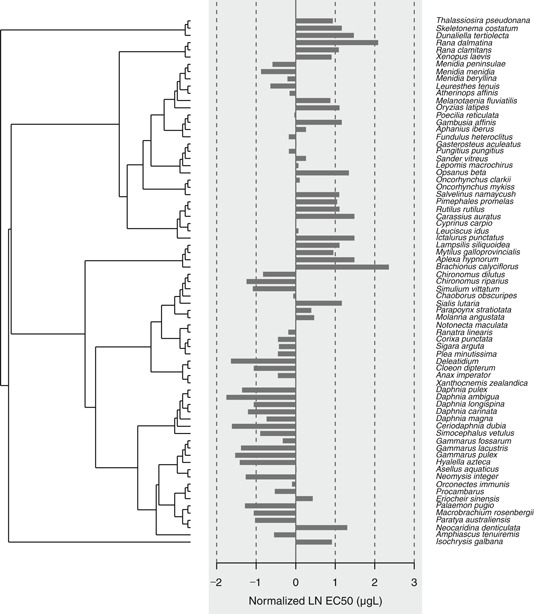
Bar plot showing normalized natural logarithm (LN) EC50 with respect to the phylogenetic tree for the chlorpyrifos all aquatic species data set. LN = natural logarithm.

**Figure 5:**
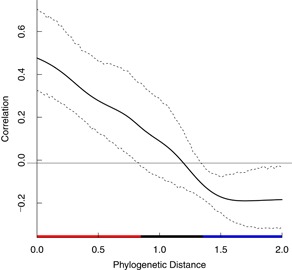
Correlogram for the chlorpyrifos all aquatic species data set. On the *x*‐axis, red indicates a positive phylogenetic autocorrelation, blue indicates a negative phylogenetic autocorrelation, and black indicates no phylogenetic autocorrelation.

**Figure 6:**
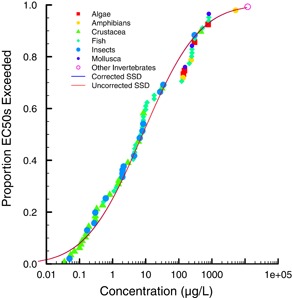
Species sensitivity distribution (SSD) for the chlorpyrifos all aquatic species data set showing the original, uncorrected SSD and the SSD corrected for phylogenetic autocorrelation.

The chlorpyrifos data set for aquatic receptors comprised 73 species. The bar plot comparing phylogeny and EC50s shows that closely related species had similar sensitivities to chlorpyrifos, whereas more distantly related species did not (Figure 4). For example, crustaceans and aquatic insects were relatively sensitive, whereas algae were not. Thus, there was a significant phylogenetic autocorrelation among the endpoints (*p* = 0.001), reducing the effective sample size from 73 to 38 (Table 1). The influence of the phylogenetic signal can also be observed in the correlogram, where both positive (between closely related species) and negative (between distantly related species) autocorrelations are present in the data set (Figure 5). The standard deviation of the original data set (3.10) and corrected data set (3.12), however, were nearly identical. Despite a significant autocorrelation, the HC5s for the original (0.0491 µg/L) and corrected (0.0475 µg/L) SSDs differed only slightly (Figure 6). The reason for this seemingly counterintuitive result is that the effective sample size is still relatively large (*n* = 38), and thus the large autocorrelation has little impact on the corrected standard deviation.

The chlorpyrifos data set was subdivided to further evaluate fish, insects, crustaceans, all invertebrates (i.e., insects, crustaceans, mollusks, and the rotifer), and all vertebrates (i.e., fish and amphibians). The insect and crustacean data sets did not produce significant phylogenetic signals (*p* = 0.0630 and 0.180, respectively), but the all vertebrates (*p* = 0.009), fish (*p* = 0.0280), and all invertebrates (*p* = 0.001) data sets did have significant phylogenetic autocorrelations (Table 1). Although the effective sample sizes were 5% to 44% lower than the numbers of species in the original data sets, the original and corrected HC5s differed by less than 5% in all subdata sets. Thus, a reduction in effective sample size had little effect on the SSDs even in the tails. See Supplemental Data Figures SI‐10 to SI‐24.

### Chlorpyrifos SSD for bird species

The chlorpyrifos SSD for birds comprised 14 species. The bar plot comparing phylogeny and oral LD50s did not show a relationship between sensitivity to chlorpyrifos and phylogeny (Supplemental Data Figure SI‐25). Thus, the phylogenetic signal was not significant (*p* = 0.178) and the effective sample size was only reduced to 13. This is also shown in the correlogram, where a significant autocorrelation was not observed (Supplemental Data Figure SI‐26). The original HC5 (8.55 mg a.i. per kilogram bw) and corrected HC5 (8.51 mg a.i. per kilogram bw) were nearly identical (Supplemental Data Figure SI‐27).

## DISCUSSION

The results indicate that the phylogenetic signal is significant for some of our toxicity data sets (i.e., the chlorpyrifos data sets for all aquatic species, all vertebrate species, all invertebrate species, and fish species) but not for others (i.e., the atrazine full and subdata sets, the chlorpyrifos data sets for all insects, crustaceans, and birds) (Table 1). Lack of a consistent phylogenetic signal in toxicity data sets was also observed by Hylton et al. (2018), who found a significant phylogenetic signal for only 10 of 42 chemicals examined for acute mortality to fish. As was the case herein with the organophosphate, chlorpyrifos, Hylton et al. (2018) did find a significant phylogenetic signal for 7 of 12 organophosphate pesticides.

Although the importance of the phylogenetic signal was mixed in the most comprehensive study done to date (Hylton et al. 2018) and in our study, other studies, many involving pesticides, have frequently observed a significant phylogenetic signal or observed that phylogeny is predictive of chemical sensitivity (e.g., Guénard et al. 2011, 2014; Hammond et al. 2012; Larras et al. 2014; Chiari et al. 2015; Keck et al. 2016; Esteves et al. 2017). In the following sections, we compare our results to other studies for atrazine and chlorpyrifos, discuss why the importance of the phylogenetic signal varies between toxicity data sets, and discuss the implications of considering phylogeny when deriving SSDs.

### Phylogenetic signal for aquatic autotrophs exposed to atrazine

Larras et al. (2014) observed a significant phylogenetic signal for 14 diatom species (i.e., super group Chromalveolata in our analyses) exposed to atrazine. We observed no phylogenetic signal for aquatic autotrophic species exposed to atrazine in spite of our data set including a broader diversity of species, that is, diatoms, autotrophic prokaryotes or blue‐green algae (Cyanobacteria), red and green algae (Archaeplastida), and flowering plants (Angiospermae). Several possible explanations exist to explain the discrepancy in results. For example, the methodology used to generate the phylogenetic trees differed between our studies. Larras et al. (2014) based their phylogenetic tree for diatoms on 2 genetic markers, 18S and rbcL, whereas our phylogenetic tree was based on names and phylogenetic lineages of more than 160 000 organisms that have molecular data in the NCBI databases. That explanation seems unlikely given that taxonomic trees based on genetic markers or other approaches are unlikely to have species switching between super groups or having species in the same genus (e.g., *Lemna minor* and *L. gibba*, *Desmodesmus subspicatus* and *D. abundans*, *Anabaena inaequalis* and *A. cylindrica*) as not being closely related (Figure 1). The more likely explanation is that the Arachaeplastida species and perhaps the Cyanobacteria species had a wide range of sensitivities to atrazine, which may have obscured the phylogenetic signal that may exist with the other super groups (Figure 1). Within the 2 subdata sets that had the largest sample sizes (Angiospermae and Archaeplastida), there is also no evidence that sensitivity to atrazine is correlated with taxonomic relatedness (Figure 1). The significant phylogenetic signal observed by Larras et al. (2014) for diatoms exposed to atrazine was also not strong, and a later study on a similar data set (i.e., Esteves et al. 2017) did not observe a significant phylogenetic signal. The importance of the phylogenetic signal for diatoms exposed to atrazine remains somewhat uncertain. We discuss another possible explanation for the discrepancy between our results and Larras et al. (2014), that is, the differing metrics using to determine phylogenetic signal, in the *Indices used to measure and test phylogenetic signal* section.

### Phylogenetic signal for aquatic species and birds exposed to chlorpyrifos

The mechanism of action for organophosphate pesticides such as chlorpyrifos is through inhibition of acetylcholinesterase (AChE) in the brain and plasma by the metabolite, chlorpyrifos oxon (Solomon et al. 2014). Organisms lacking the target enzyme (e.g., aquatic plants and algae) are relatively insensitive to chlorpyrifos exposure (Giddings et al. 2014). Although chlorpyrifos is toxic to most animal species, there is a wide range of sensitivities due to differences in toxicokinetics between species (i.e., adsorption, distribution, metabolism, and excretion) (Solomon et al. 2014). Our analyses indicated a strong phylogenetic signal for the data set that included all aquatic taxonomic groups (including aquatic plants) and a somewhat weaker but significant signal for 3 of the subdata sets (i.e., all vertebrates, all invertebrates, fish). The phylogenetic signal was weak and not significant in the subdata sets for insects, crustaceans, and birds) (Table 1). Of the toxicity data sets that we considered, the all aquatic species data set for chlorpyrifos had by far the widest range of sensitivities with a 148 000‐fold difference in toxicity between the most and least sensitive species. By comparison, the ranges in sensitivities for all aquatic autotrophs exposed to atrazine and birds exposed to chlorpyrifos were 47‐fold and 22‐fold, respectively. Thus, it appears that a stronger phylogenetic signal occurs in data sets that include a broader array of taxonomic groups and a wide sensitivity range.

### Indices used to measure and test phylogenetic signal

Phylogenetic signal is the tendency of related species to resemble one another with regard to a trait (e.g., sensitivity to a pesticide) more so than would species drawn at random from the same phylogenetic tree (Blomberg and Garland 2002; Blomberg et al. 2003). Various indices have been proposed and used for quantifying strength of the phylogenetic signal, sometimes leading to contrasting results (Münkemüller et al. 2012). Several indices have been adapted from a spatial autocorrelation context (e.g., Moran's *I*, Abouheif's *C*_mean_) and are not based on an assumed evolutionary model (Münkemüller et al. 2012). Others such as Pagel's λ and Blomberg's *K* explicitly relate to a Brownian motion (BM) model of evolution (Blomberg et al. 2003; Münkemüller et al. 2012). These 4 indices capture different aspects of the phylogenetic signal, and each has their advantages and disadvantages (see Revell et al. 2008 and Münkemüller et al. 2012 for a more detailed discussion). For example, the autocorrelation indices have better robustness to inaccurate phylogenetic information and impose less restrictive assumptions. These indices, however, are less suitable for inferring evolutionary processes than are the BM indices or other indices that assume an underlying evolutionary model. We used Moran's *I* to determine phylogenetic signal, primarily because the resulting autocorrelation parameter could be used to determine effective sample size (see Eqn. 2). Effective sample size is a necessary input in deriving lognormal SSDs that correct for the phylogenetic signal. Moran's *I* can also be used to generate correlograms that illustrate the relationship between autocorrelation with regard to sensitivity and phylogenetic distance (Keck et al. 2016).

The magnitudes of the values from the different indices are not immediately comparable because they have different ranges (i.e., –1 to 1 for Abouheif's *C*_mean_ and Moran's *I*, 0 to >1 for Pagel's λ and Blomberg's *K*). Each index can, however, be tested for the absence of a phylogenetic signal, that is, when sensitivity is randomly distributed in the phylogeny. Our results indicate that the absence or presence of a significant phylogenetic signal was consistent across the indices for all but 2 of the 10 toxicity data sets, that is, insects and fish exposed to chlorpyrifos (Table 2). For the latter 2 data sets, the significance or lack of significance of the phylogenetic signal depended on which index was used.

**Table 2:** Comparison of indices for phylogenetic signal

Parameter	Atrazine			Chlorpyrifos						
	All aquatic autotrophs	Angiospermae	Archaeplastida	All aquatic receptors	All vertebrates	All invertebrates	Fish	Insects	Crustaceans	Birds
Abouheif's *C*_mean_	0.153	–0.334	–0.0806	0.563	0.339	0.469	0.252	0.302	0.0645	0.142
Moran's *I*	0.0653	–0.214	–0.111	0.318	0.183	0.293	0.129	0.112	0.0129	0.0499
Blomberg's *K*	0.144	0.273	0.227	0.180	0.222	0.238	0.264	0.525	0.165	0.271
Pagel's λ	0.207	7.56E–05	7.16E–05	0.757	0.449	0.753	0.342	1.01	0.128	7.16E–05
Abouheif's *C*_mean_ (*p*‐value)	0.077	0.926	0.473	0.001	0.006	0.001	0.023	0.016	0.174	0.158
Moran's *I* (*p*‐value)	0.077	0.767	0.551	0.001	0.009	0.001	0.028	0.063	0.180	0.178
Blomberg's *K* (*p*‐value)	0.070	0.691	0.498	0.001	0.007	0.001	0.017	0.003	0.544	0.158
Pagel's λ (*p*‐value)	0.244	1	1	0.001	0.044	0.001	0.208	0.0388	0.506	1

### Implications of phylogenic autocorrelation on derivation of SSDs

Despite strong phylogenetic autocorrelations in several of our toxicity data sets, the SSDs and resulting HC5s were scarcely affected. The lack of effect on the HC5s was in spite of large declines between original sample size and effective sample size following correction for a significant phylogenetic signal (e.g., original *n* = 73, *n*_eff_ = 38 for the chlorpyrifos all aquatic species data set). Even with the reduced sample size in the 2 largest chlorpyrifos data sets that had a significant phylogenetic signal (i.e., all aquatic biota, all invertebrate species), effective sample size was still relatively large (i.e., 38 and 23, respectively; Table 1), thus minimizing the impact on the variance estimator for the lognormal distribution.

It is possible that the HC5 would be more strongly impacted with smaller sample size data sets (*n* < 15) that have a strong phylogenetic signal, a scenario that did not arise in our case study data sets. To investigate this possibility, we created 2 small hypothetical data sets that included a wide range of sensitivities and taxonomic relatedness but had a significant phylogenetic signal. For the first hypothetical small data set, we selected 2 species from each of the crustacean, insect, mollusk, fish, amphibian, and algal taxonomic groups plus the 1 rotifer species (i.e., *Brachionus calyciflorus*) from the all aquatic biota toxicity data set for chlorpyrifos (Table 3). To ensure a reasonably strong phylogenetic signal, we selected the 2 species closest to the median toxicity value within each taxonomic group. The results indicated a significant phylogenetic signal (Moran's *I* = 0.236, *p* = 0.013), but the HC5 values changed only slightly between the uncorrected SSD (i.e., 0.151 μg/L) and the SSD corrected for the phylogenetic signal (i.e., 0.132 μg/L) (see Supplemental Data Figures SI‐27 to SI‐30). This was in spite of a reduction from the original sample size of 13 to an effective sample size of 8.29. For the second hypothetical small data set, we wanted to create as strong a phylogenetic signal as possible. This task was done by “cherry picking” species from the chlorpyrifos data set for all aquatic biota such that sensitivity similarity was strongly correlated with taxonomic relatedness. For this data set, we selected 3 species each from the crustacean, insect, fish, and algal taxonomic groups (Table 4). The results indicated a strongly significant phylogenetic signal (Moran's *I* = 0.378, *p* = 0.001), but the HC5 values changed only modestly between the uncorrected SSD (i.e., 0.0515 μg/L) and the SSD corrected for the phylogenetic signal (i.e., 0.0391 μg/L) (see Supplemental Data Figures SI‐31 to SI‐33). This was in spite of a reduction from the original sample size of 12 to an effective sample size of 5.68.

**Table 3:** Input data for first hypothetical chlorpyrifos aquatic data set with a reduced sample size

Taxon	Species name	NCBI species name	Taxonomic ID	Geomean effect concentration (μg/L)
Alga	*Skeletonema costatum*	*Skeletonema costatum*	2843	298
Alga	*Thalassiosira pseudonana*	*Thalassiosira pseudonana*	35 128	148
Amphibian	*Lithobates clamitans clamitans*	*Rana clamitans*	145 282	236
Amphibian	*Rana dalmatina*	*Rana dalmatina*	51 331	5174
Crustacean	*Daphnia longispina*	*Daphnia longispina*	42 846	0.3
Crustacean	*Macrobrachium rosenbergii*	*Macrobrachium rosenbergii*	79 674	0.3
Fish	*Lepomis macrochirus*	*Lepomis macrochirus*	13 106	10
Fish	*Leuciscus idus*	*Leuciscus idus*	69 811	10
Insect	*Corixa punctata*	*Corixa punctata*	1 545 103	2
Insect	*Sigara arguta*	*Sigara arguta*	489 481	2.16
Mollusk	*Lampsilis siliquoidea*	*Lampsilis siliquoidea*	52 396	250
Mollusk	*Mytilus galloprovincialis*	*Mytilus galloprovincialis*	29 158	154
Rotifer	*Brachionus calyciflorus*	*Brachionus calyciflorus*	104 777	12 000

ID = identifier; NCBI = National Center for Biotechnology Information (US).

**Table 4:** Input data for second hypothetical chlorpyrifos aquatic data set with a reduced sample size

Taxon	Species name	NCBI species name	Taxonomic ID	Geomean effect concentration (μg/L)
Crustacean	*Ceriodaphnia dubia*	*Ceriodaphnia dubia*	117 530	0.054
Crustacean	*Daphnia carinata*	*Daphnia carinata*	120 202	0.19
Crustacean	*Daphnia longispina*	*Daphnia longispina*	42 846	0.3
Insect	*Corixa punctata*	*Corixa punctata*	1 545 103	2
Insect	*Sigara arguta*	*Sigara arguta*	489 481	2.16
Insect	*Notonecta maculata*	*Notonecta maculata*	1 545 171	7.97
Fish	*Lepomis macrochirus*	*Lepomis macrochirus*	13 106	10
Fish	*Sander vitreus*	*Sander vitreus*	283 036	18
Fish	*Melanotaenia fluviatilis*	*Melanotaenia fluviatilis*	120 844	122
Alga	*Thalassiosira pseudonana*	*Thalassiosira pseudonana*	35 128	148
Alga	*Skeletonema costatum*	*Skeletonema costatum*	2843	298
Alga	*Dunaliella tertiolecta*	*Dunaliella tertiolecta*	3047	769

ID = identifier; NCBI = National Center for Biotechnology Information (US).

Wheeler et al. (2002) estimated that a minimum sample size of 10 to 15 is required to derive a stable estimate of endpoints such as the HC5 (see also Pennington 2003). With the exception of our 2 hypothetical data sets, the effective sample sizes of our data sets for atrazine and chlorpyrifos after correction for the phylogenetic signal ranged from 13 to 38. Thus, our HC5 estimates remained stable even with the reduced effective sample sizes.

Even if the HC5 values are corrected for the bias that results from reduced effective sample sizes using the extrapolation factors derived by Aldenberg and Jaworska (2000), the HC5 values are only slightly affected. Consider the chlorpyrifos data set for aquatic insects, which had the smallest effective sample size outside of the hypothetical data sets. The mean (i.e., the HC50) and standard deviation for the fitted lognormal distribution were 2.59 and 2.21, respectively (Table 1). Applying the formula from Aldenberg and Jaworska (2000) without correcting for sample size (their Equation 2, i.e., log(HC5) = log(HC50) – 1.6449 × *s*, where *s* is the standard deviation) results in an estimated median HC5 of 0.068 μg/L, which is the same as the uncorrected HC5 shown in Table 1. When the median HC5 is corrected for the effective sample size of 13 and corrected standard deviation of 2.23 using the extrapolation factor (*k*_*s*_) of 1.687 from Aldenberg and Jaworska (2000; see their Table 1), the resulting HC5 is only slightly reduced, 0.060 μg/L. The bias correction is even smaller for the other original data sets (Table 1). Thus, for our original data sets, the bias resulting from reduced effective sample size does not change our conclusion that the phylogenetic signal, even when it is significant, has only a minor impact on estimated HC5 values from SSDs. However, if we consider the smaller hypothetical data sets that had a strong phylogenetic signal (Tables 3 and 4), the importance of the bias arising from a reduced effective sample size becomes more important. For the first hypothetical data set (Table 3), the HC5 corrected for the sample size bias per Aldenberg and Jaworska (2000) is 0.105 μg/L, which is below the HC5 corrected for the phylogenetic signal of 0.132 μg/L and the HC5 not corrected for the phylogenetic signal of 0.151 μg/L. The corresponding HC5 values for the second hypothetical data set (Table 4) are 0.0269 μg/L after correction for sample size bias, 0.0391 μg/L after correction for the phylogenetic signal, and 0.0515 μg/L without a correction for phylogenetic signal. Thus, in extreme situations (i.e., small data sets with very strong phylogenetic signals), HC5 values may be significantly reduced if the HC5 is corrected for sample size bias. That said, the reduction is less than 2‐fold even in those extreme situations.

## CONCLUSIONS

Species sensitivity distributions are now commonly used in ecological risk assessments and to derive environmental quality benchmarks. There are, however, several questionable statistical and ecological assumptions underlying derivation of SSDs. One key statistical assumption is that the input data are iid, a tenuous assumption given that closely related species are more likely to have similar chemical sensitivities than are distantly related species. There is now a growing literature indicating that the assumption of independence is not met in many multispecies toxicity data sets, that is, there is a significant phylogenetic signal (e.g., Guénard et al. 2011, 2014; Hammond et al. 2012; Larras et al. 2014; Chiari et al. 2015; Keck et al. 2016; Esteves et al. 2017; Hylton et al. 2018). We also observed a significant phylogenetic signal in several of our chlorpyrifos toxicity data sets although not in our atrazine toxicity data sets. When a significant phylogenetic signal did occur, the result was a reduced effective sample size to account for lack of independence between data points. The reduction was rather large when the signal was strong. In spite of the reduced effective sample sizes, significant phylogenetic signals had little impact on fitted SSDs, even in the tails (e.g., HC5 values). The impact of the phylogenetic signal became important only when we artificially reduced original sample size, increased strength of the phylogenetic signal, and corrected for sample size bias per Aldenberg and Jaworska (2000). We conclude that it is good statistical practice to account for the phylogenetic signal when deriving SSDs because many multispecies toxicity data sets do not meet the independence assumption. That said, SSDs and HC5 values appear to be robust to deviations from the independence assumption.

## Disclaimer

The peer‐review process for this article was managed by the Editorial Board without the involvement of RA Brain.

## SUPPLEMENTAL DATA

Includes all input data sets and outputs not included in main manuscript. Also includes example R code used to conduct statistical analyses.

**Figure SI‐1**. Phylogenetic tree for the atrazine all aquatic plant species data set.

**Figure SI‐2**. Phylogenetic tree for the chlorpyrifos all aquatic species data set.

**Figure SI‐3**. Phylogenetic tree for the chlorpyrifos all bird species data set.

**Figure SI‐4**. Bar plot showing normalized LN EC50 with respect to phylogenetic tree for the atrazine Angiospermae data set.

**Figure SI‐5**. Correlogram for the atrazine Angiospermae data set.

**Figure SI‐6**. SSDs for the atrazine Angiospermae data set showing original and corrected model fits.

**Figure SI‐7**. Bar plot showing normalized LN EC50 with respect to phylogenetic tree for the atrazine Archaeplastida data set.

**Figure SI‐8**. Correlogram for the atrazine Archaeplastida data set.

**Figure SI‐9**. SSDs for the atrazine Archaeplastida data set showing original and corrected model fits.

**Figure SI‐10**. Bar plot showing normalized LN EC50 with respect to phylogenetic tree for the chlorpyrifos all aquatic vertebrates data set.

**Figure SI‐11**. Correlogram for the chlorpyrifos all aquatic vertebrates data set.

**Figure SI‐12**. Species sensitivity distributions for the chlorpyrifos all aquatic vertebrates data set showing original and corrected model fits.

**Figure SI‐13**. Bar plot showing normalized LN EC50 with respect to phylogenetic tree for the chlorpyrifos all aquatic invertebrates data set.

**Figure SI‐14**. Correlogram for the chlorpyrifos all aquatic invertebrates data set.

**Figure SI‐15**. Species sensitivity distributions for the chlorpyrifos all aquatic invertebrates data set showing original and corrected model fits.

**Figure SI‐16**. Bar plot showing normalized LN EC50 with respect to phylogenetic tree for the chlorpyrifos fish data set.

**Figure SI‐17**. Correlogram for the chlorpyrifos fish data set.

**Figure SI‐18**. Species sensitivity distributions for the chlorpyrifos fish data set showing original and corrected model fits.

**Figure SI‐19**. Bar plot showing normalized LN EC50 with respect to phylogenetic tree for the chlorpyrifos aquatic insects data set.

**Figure SI‐20**. Correlogram for the chlorpyrifos aquatic insects data set.

**Figure SI‐21**. SSDs for the chlorpyrifos aquatic insects data set showing original and corrected model fits.

**Figure SI‐22**. Bar plot showing normalized LN EC50 with respect to phylogenetic tree for chlorpyrifos crustaceans data set.

**Figure SI‐23**. Correlogram for chlorpyrifos crustaceans data set.

**Figure SI‐24**. Species sensitivity distribution for chlorpyrifos crustaceans data set showing original and corrected model fit.

**Figure SI‐25**. Bar plot showing normalized LN EC50 with respect to phylogenetic tree for chlorpyrifos birds data set.

**Figure SI‐26**. Correlogram for chlorpyrifos birds data set.

**Figure SI‐27**. Species sensitivity distribution for chlorpyrifos birds data set showing original and corrected model fit.

**Figure SI‐28**. Bar plot showing normalized LN EC50 with respect to phylogenetic tree for first hypothetical chlorpyrifos aquatic data set with a reduced sample size.

**Figure SI‐29**. Correlogram for first hypothetical chlorpyrifos aquatic data set.

**Figure SI‐30**. Species sensitivity distribution for first hypothetical chlorpyrifos aquatic data set showing original and corrected model fit.

**Figure SI‐31**. Bar plot showing normalized LN EC50 with respect to phylogenetic tree for second hypothetical chlorpyrifos aquatic data set with a reduced sample size.

**Figure SI‐32**. Correlogram for second hypothetical chlorpyrifos aquatic data set.

**Figure SI‐33**. Species sensitivity distribution for second hypothetical chlorpyrifos aquatic data set showing original and corrected model fit.

**Table SI‐1**. Input data for atrazine aquatic plants data set (USEPA 2012)

**Table SI‐2**. Input data for chlorpyrifos aquatic species data set (Giddings et al. 2014)

**Table SI‐3**. Input data for chlorpyrifos birds data set (Moore et al. 2014)

**Example R Code** (chlorpyrifos data set for all aquatic species)

## Supporting information

This article contains online‐only Supplemental Data.

Supplementary information.

## Data Availability

All original input data files and outputs not shown in the main body of the manuscript are available in a Supplemental Data file. The Supplemental Data file also includes an example of the R code used to conduct the statistical analyses.
