# Supplementary material for: Correcting for Phylogenetic Autocorrelation in Species Sensitivity Distributions
Source: Integr Environ Assess Manag. 2019 Nov 18;16(1):53–65. doi: 10.1002/ieam.4207 (PMC6972980; doi:10.1002/ieam.4207)
Supplement: Supplementary file 1 — Supplementary information. [file IEAM-16-53-s001.pdf]

Supplemental Information

Figures

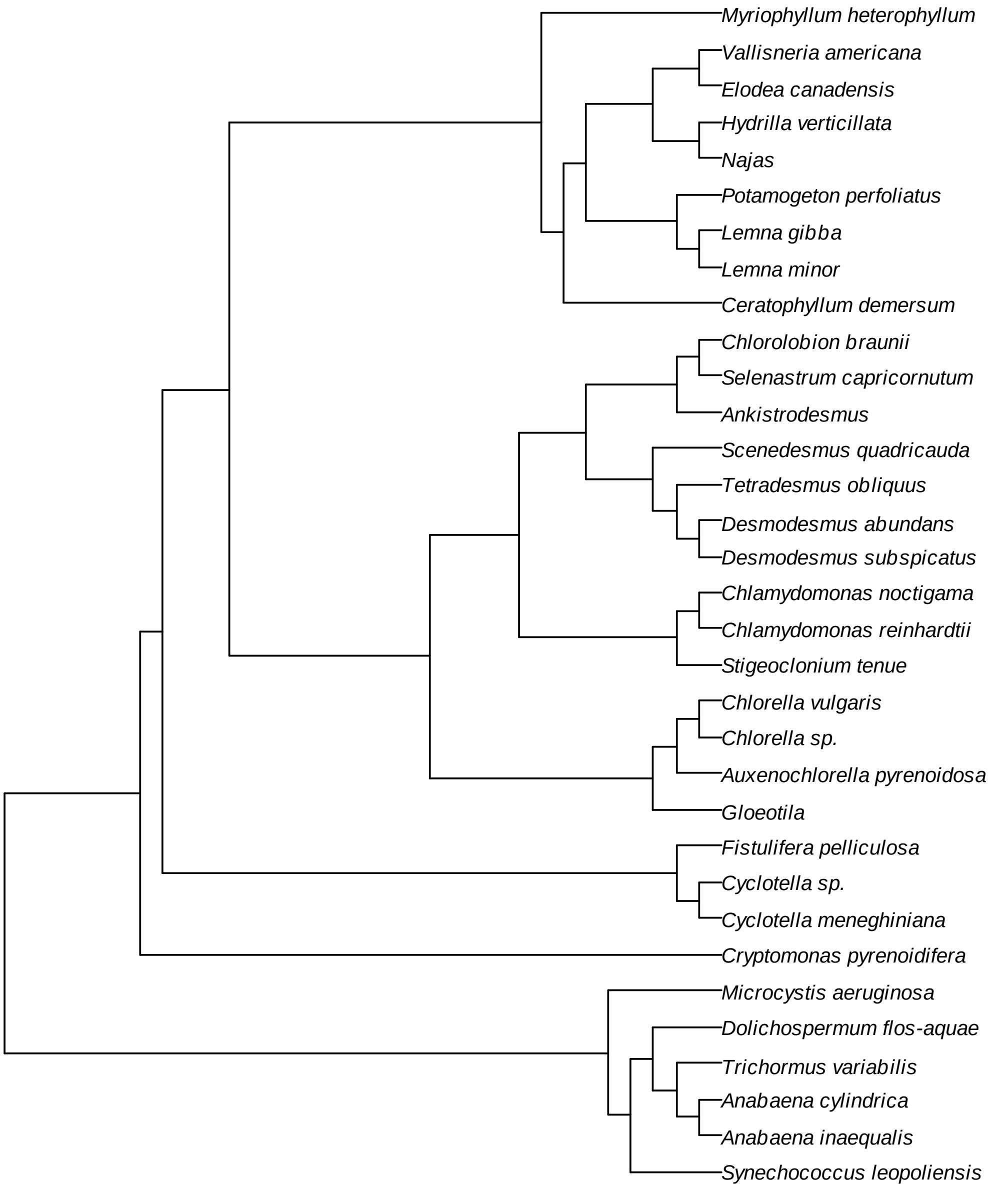

Figure SI-1 Phylogenetic tree for the atrazine all aquatic plant species dataset.

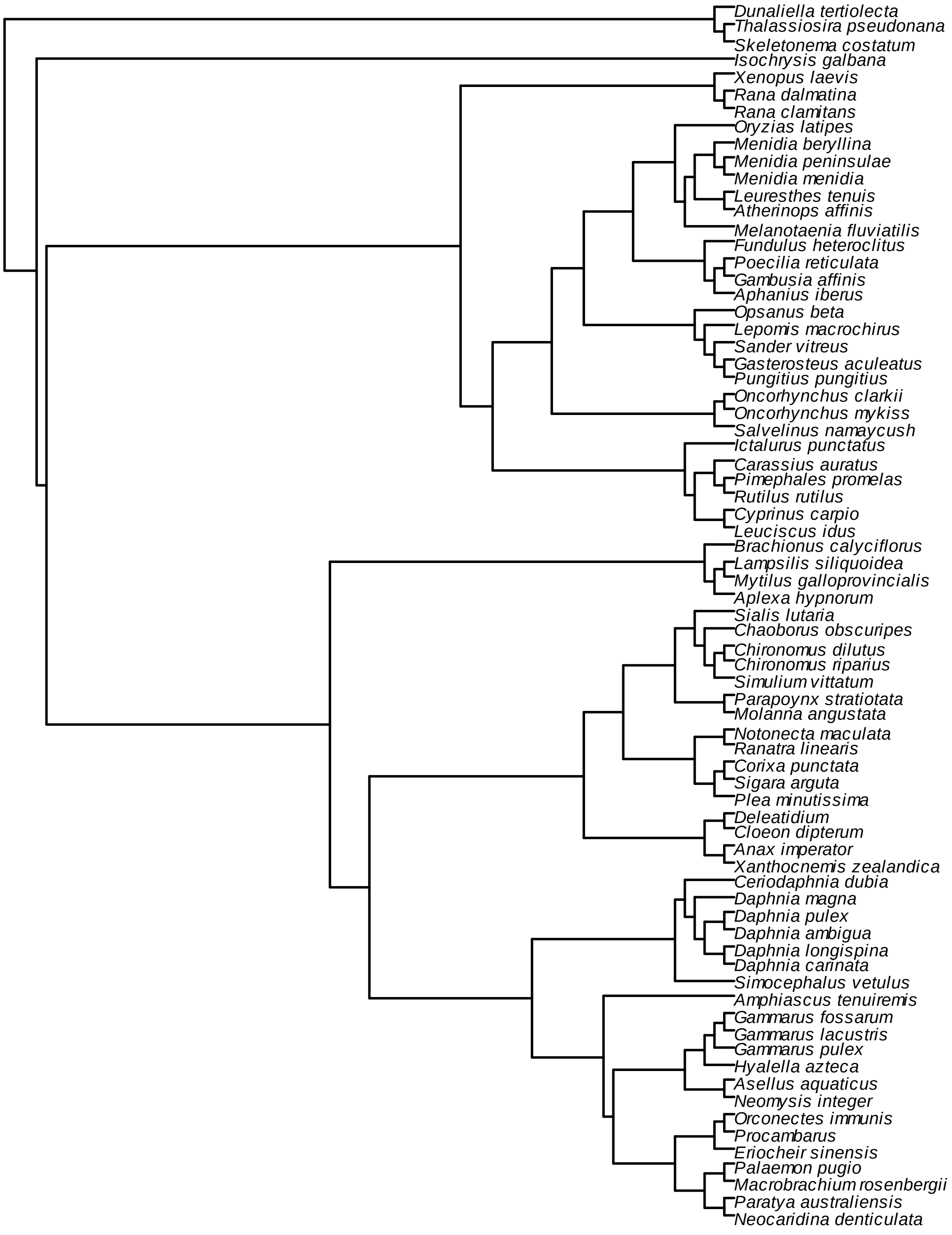

Figure SI-2 Phylogenetic tree for the chlorpyrifos all aquatic species dataset.

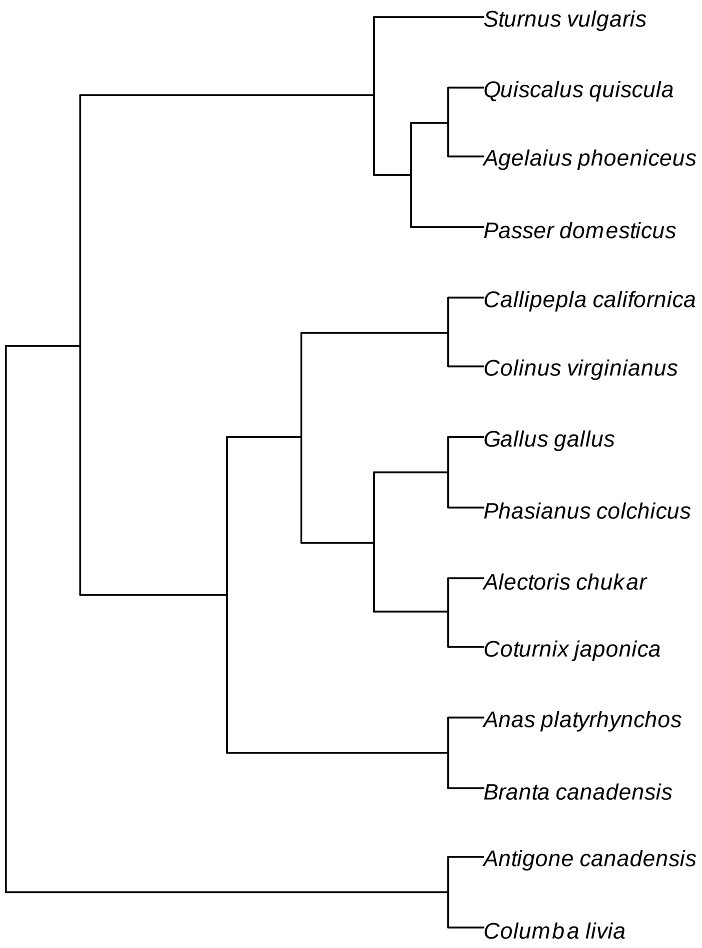

Figure SI-3 Phylogenetic tree for the chlorpyrifos all bird species dataset.

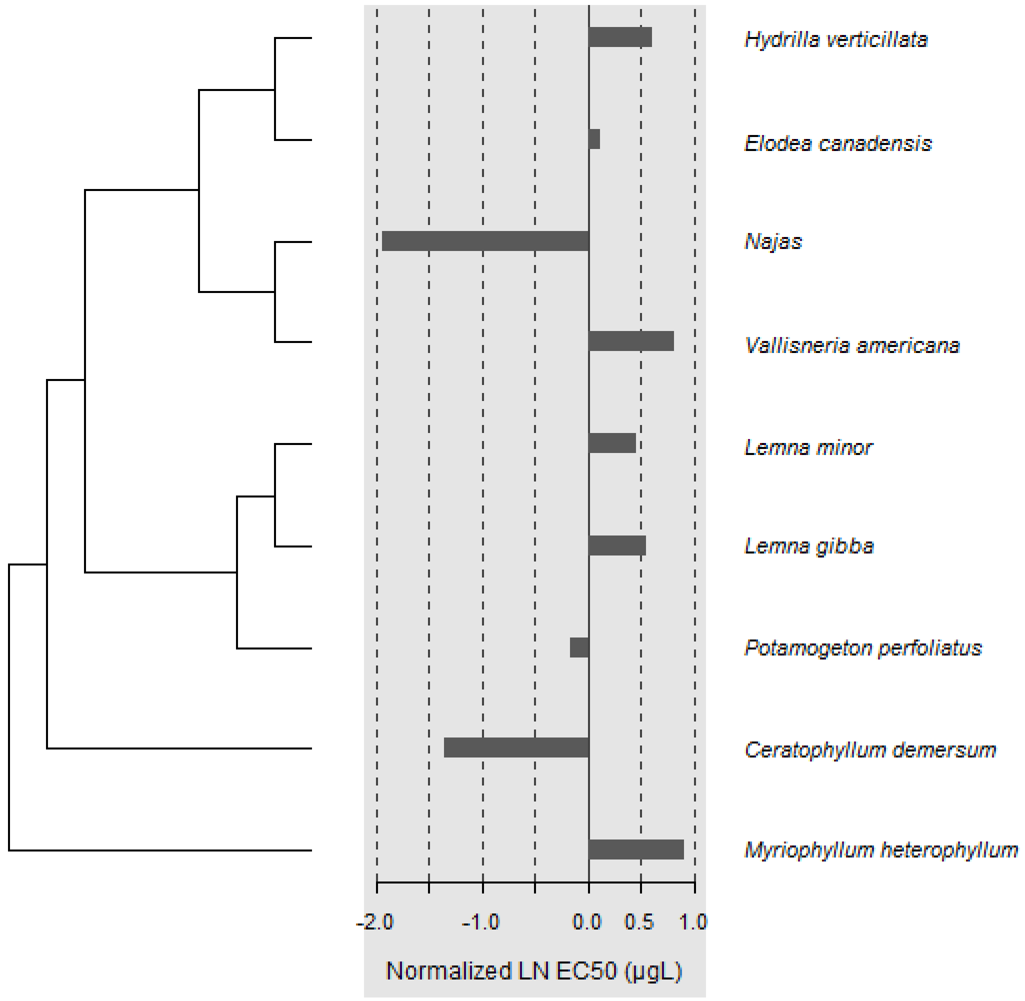

Figure SI-4 Bar plot showing normalized LN EC50 (i.e., median LN EC50=0) with respect to phylogenetic tree for the atrazine Angiospermae dataset.

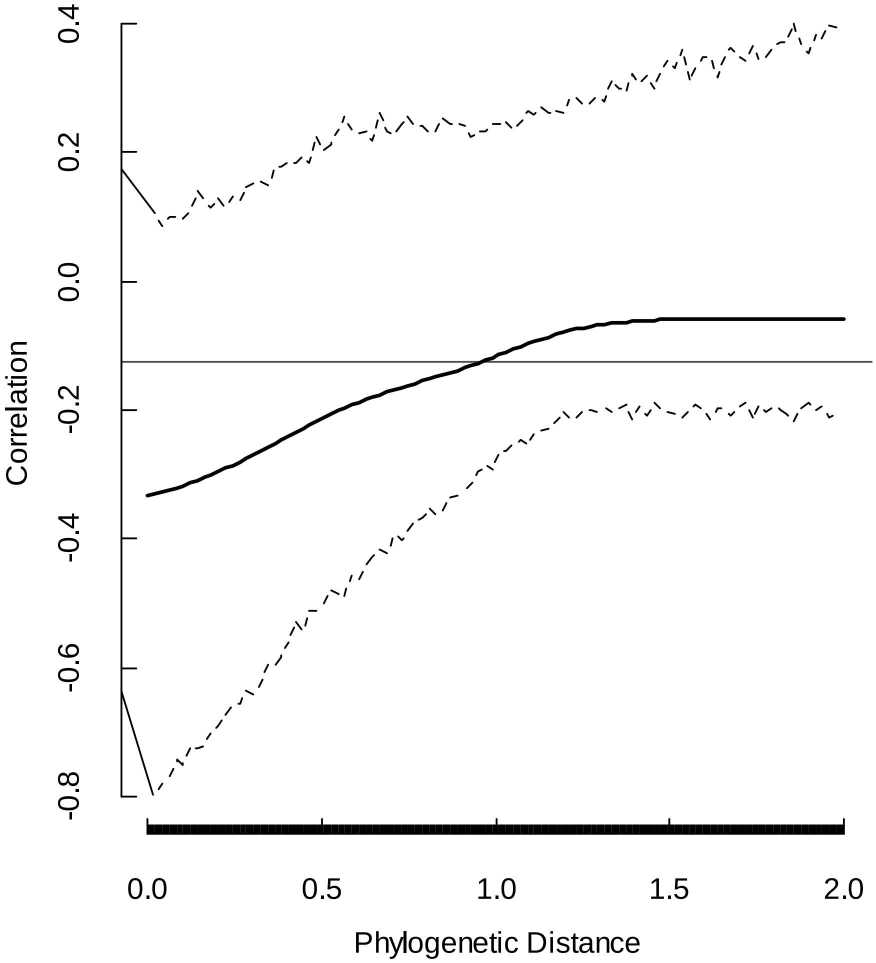

Figure SI-5 **Correlogram for the atrazine Angiospermae dataset. On the x-axis, red indicates a positive phylogenetic autocorrelation and black indicates no phylogenetic autocorrelation.**

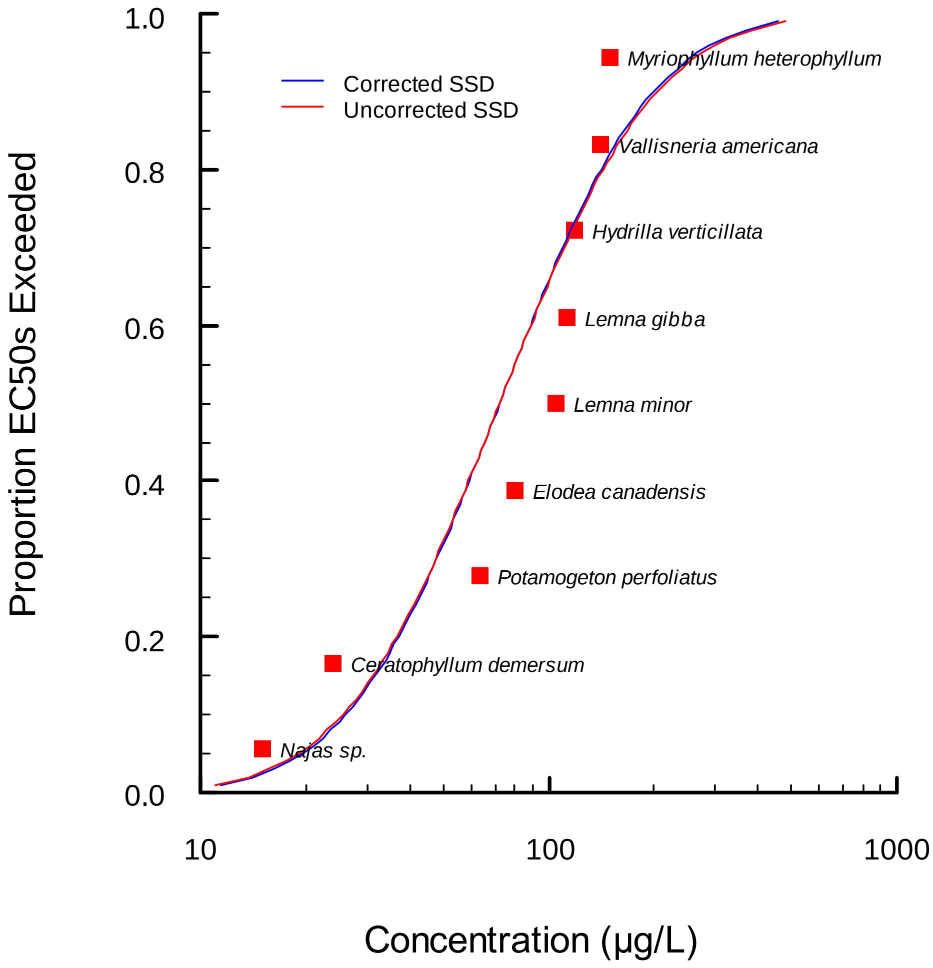

Figure SI-6 SSDs for the atrazine Angiospermae dataset showing original and corrected model fits.

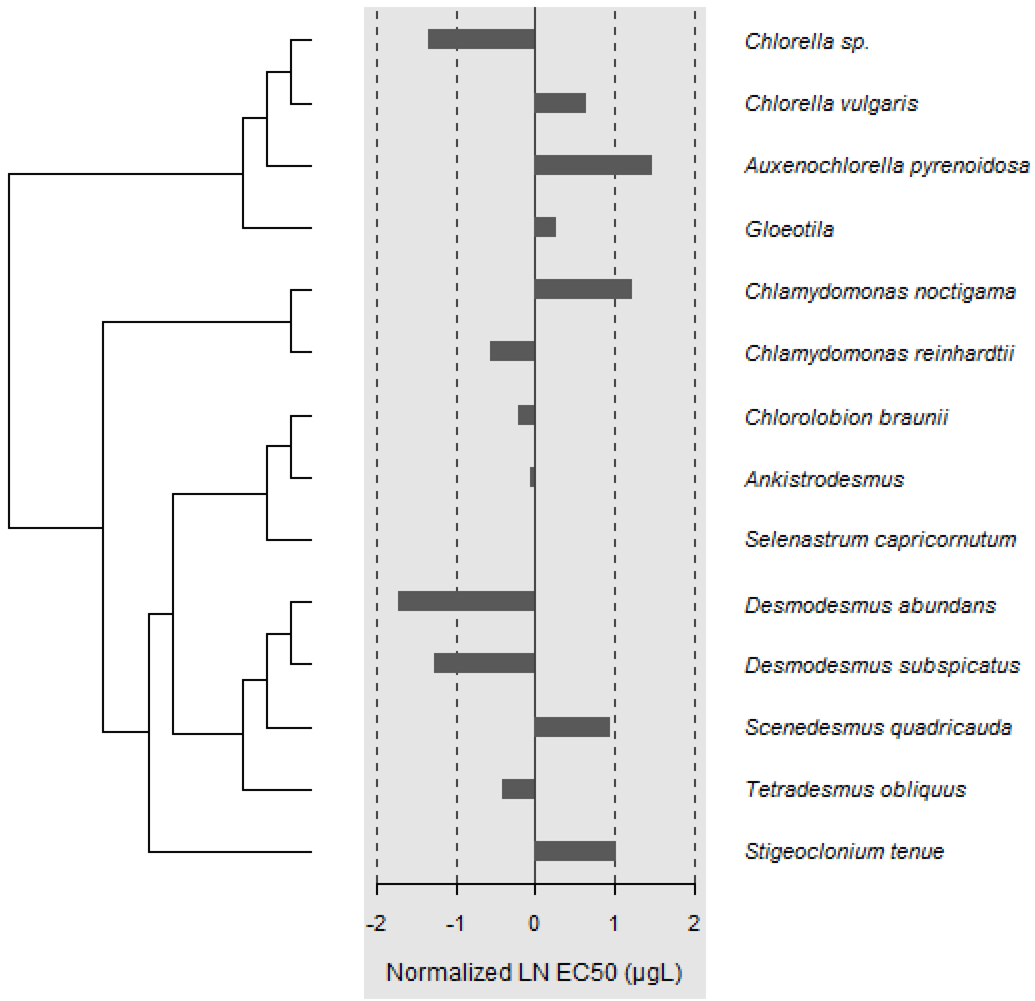

Figure SI-7 Bar plot showing normalized LN EC50 (i.e., median LN EC50=0) with respect to phylogenetic tree for the atrazine Archaeplastida dataset.

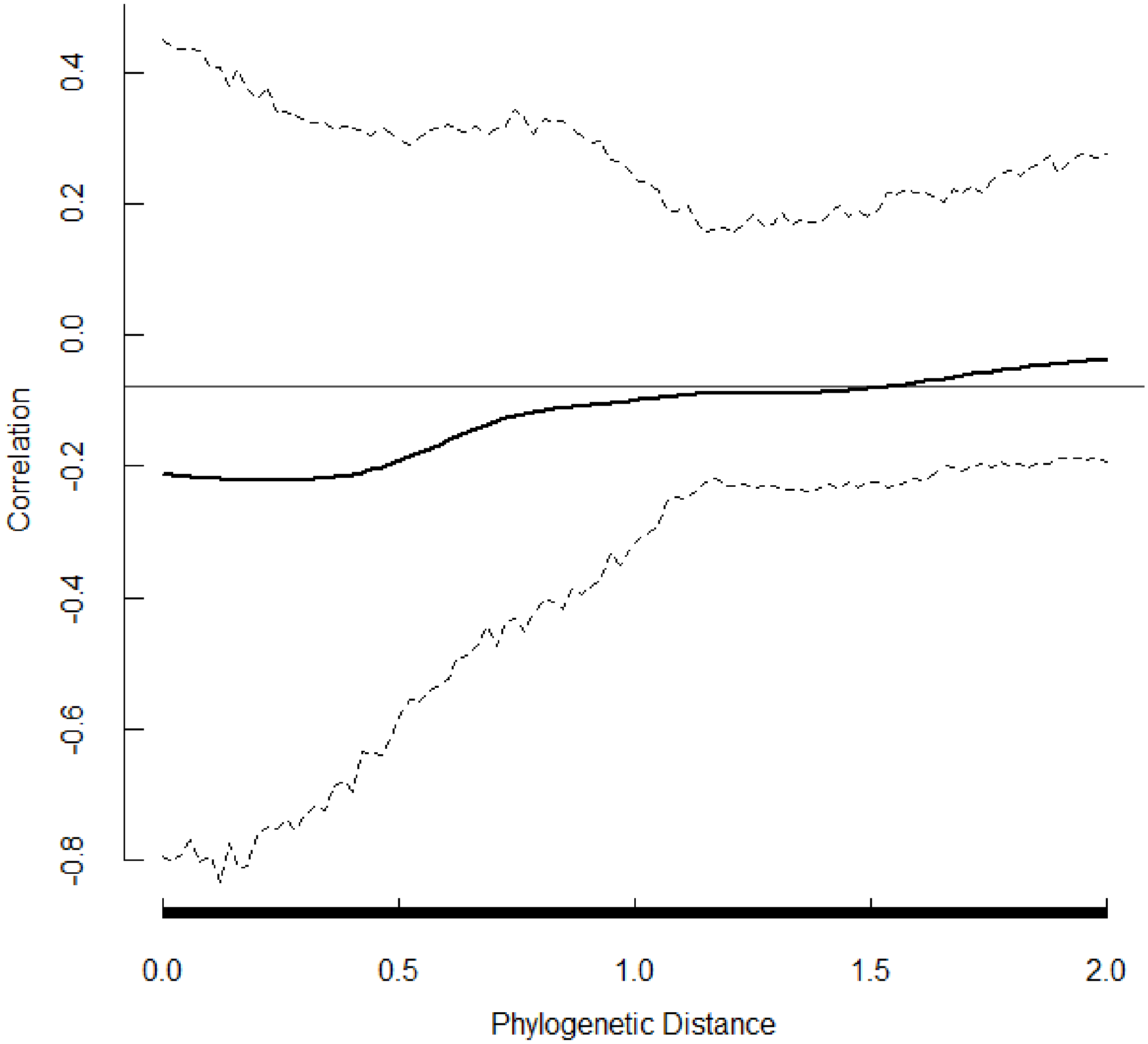

Figure SI-8 **Correlogram for the atrazine Archaeplastida dataset. On the x-axis, red indicates a positive phylogenetic autocorrelation and black indicates no phylogenetic autocorrelation.**

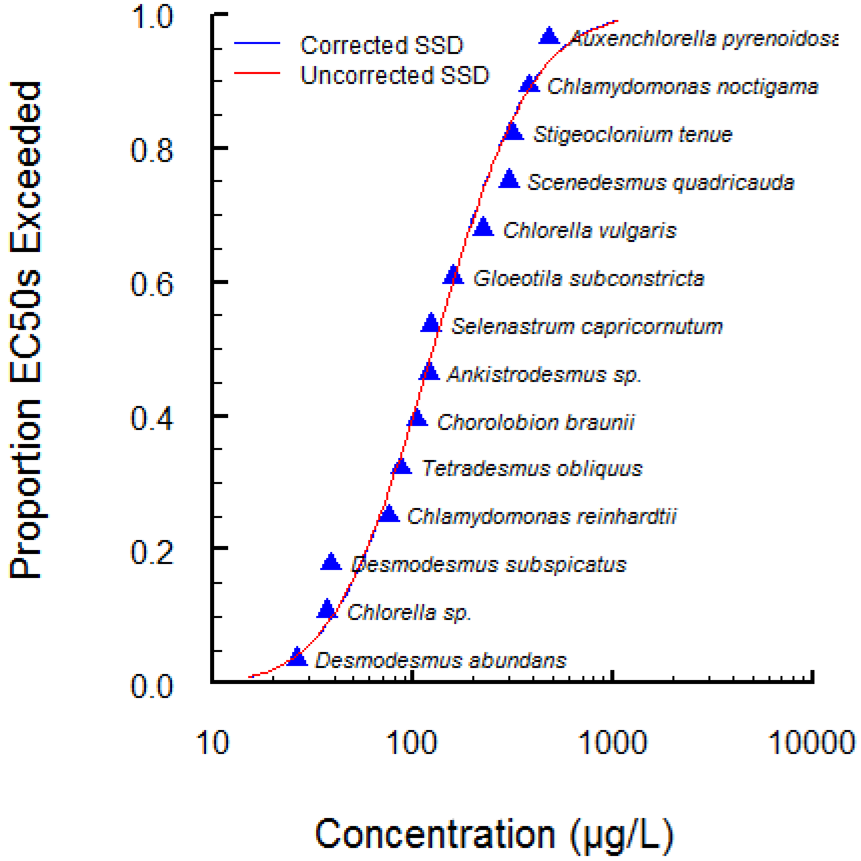

Figure SI-9 SSDs for the atrazine Archaeplastida dataset showing original and corrected model fits.

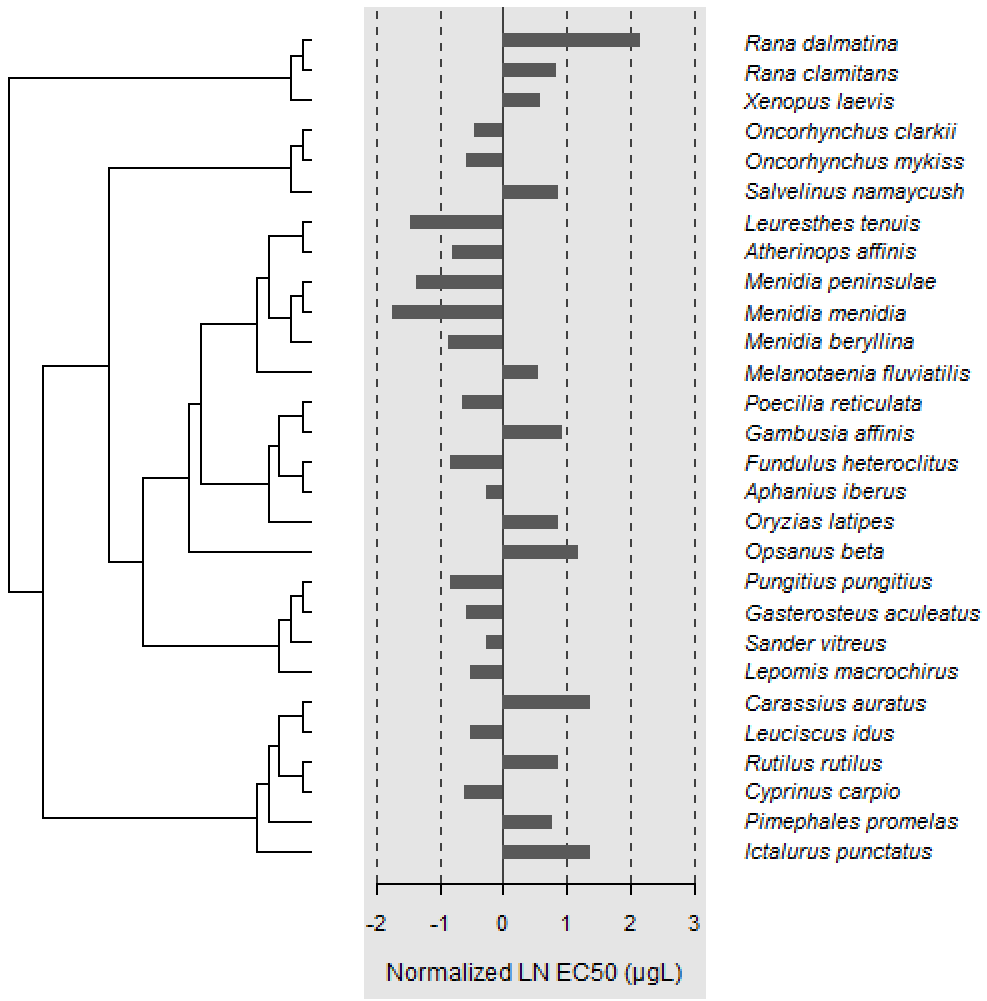

Figure SI-10 Bar plot showing normalized LN EC50 (i.e., median LN EC50=0) with respect to phylogenetic tree for the chlorpyrifos all aquatic vertebrates dataset.

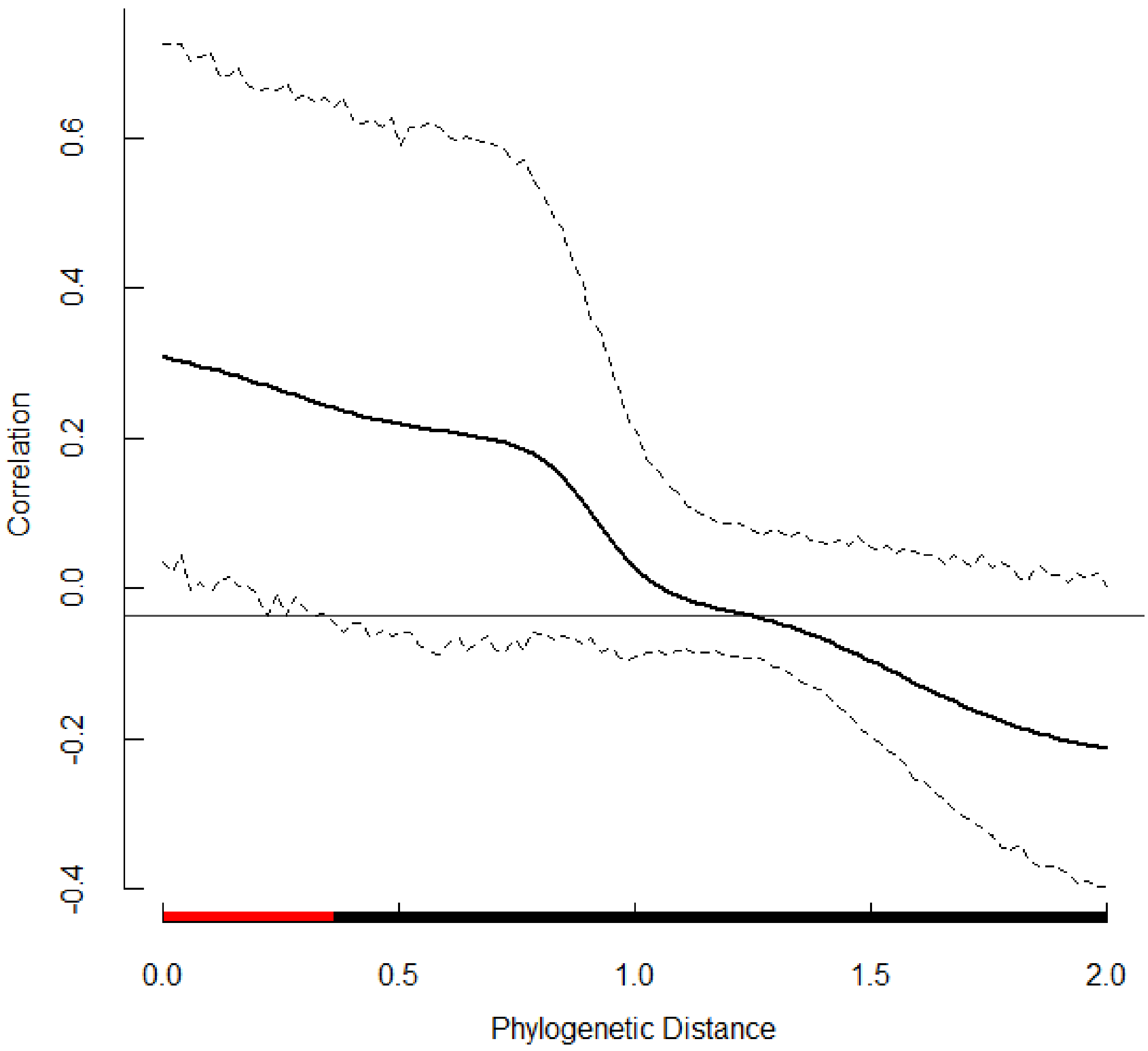

Figure SI-11 **Correlogram for the chlorpyrifos all aquatic vertebrates dataset. On the x-axis, red indicates a positive phylogenetic autocorrelation and black indicates no phylogenetic autocorrelation.**

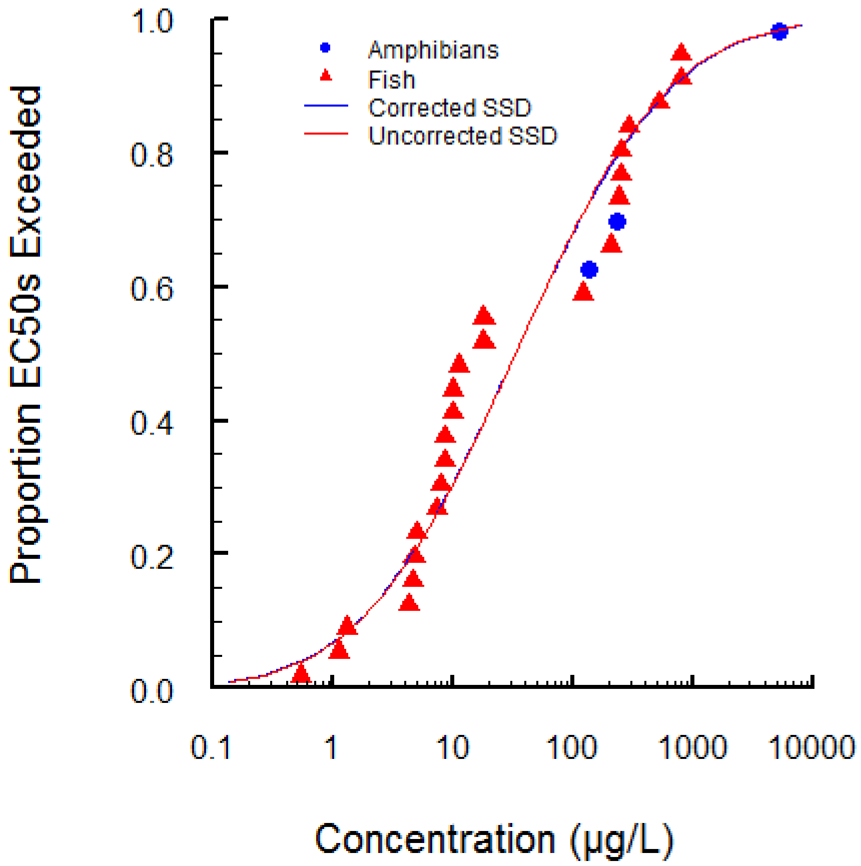

Figure SI-12 SSDs for the chlorpyrifos all aquatic vertebrates dataset showing original and corrected model fits.

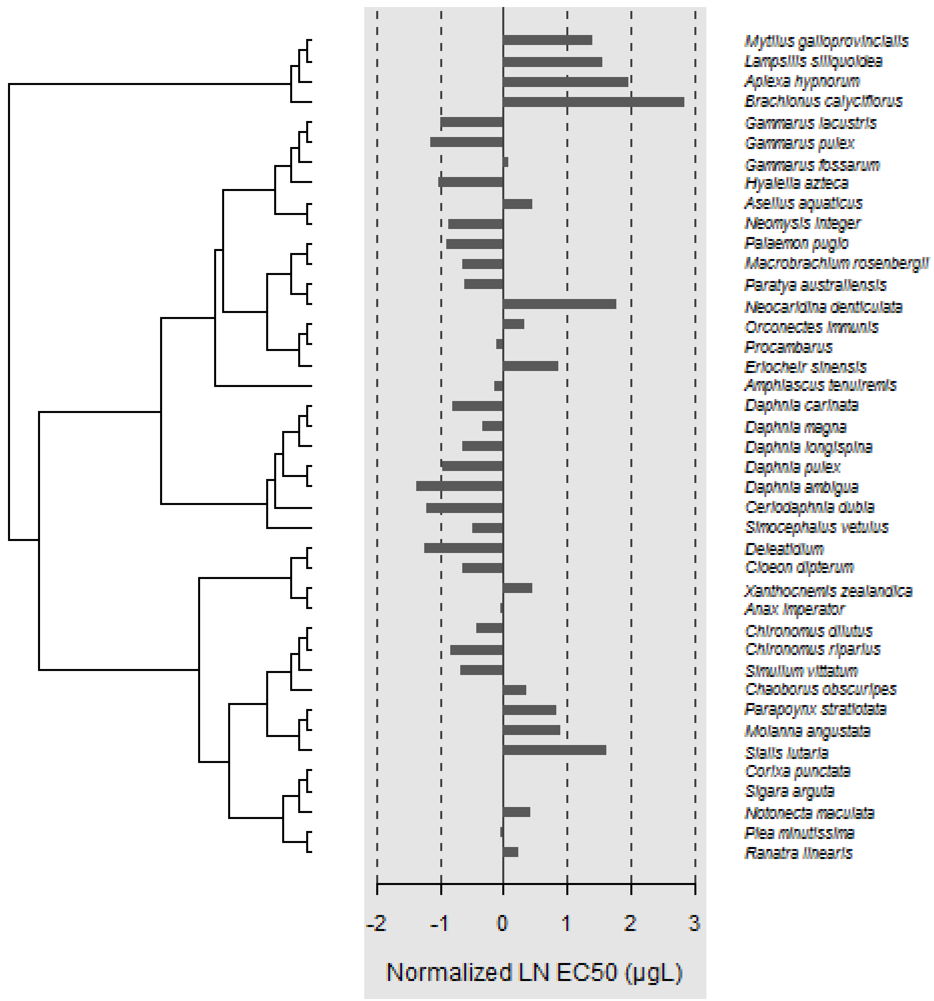

Figure SI-13 Bar plot showing normalized LN EC50 (i.e., median LN EC50=0) with respect to phylogenetic tree for the chlorpyrifos all aquatic invertebrates dataset.

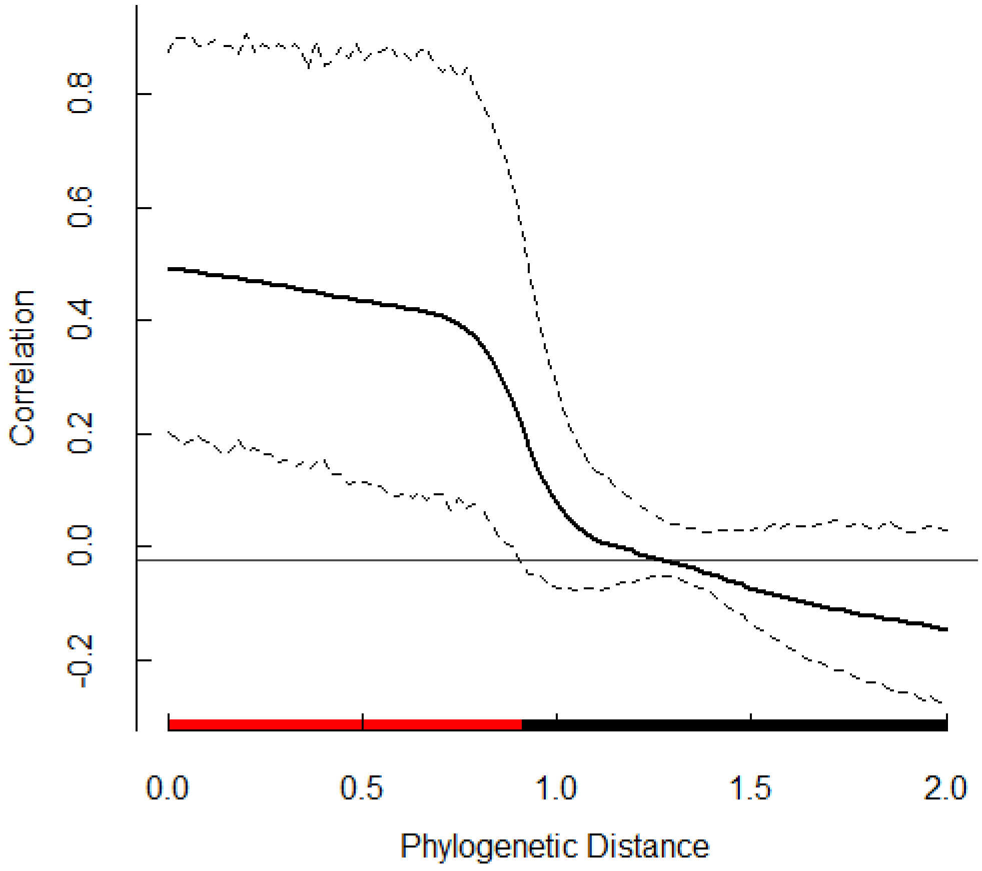

Figure SI-14 **Correlogram for the chlorpyrifos all aquatic invertebrates dataset. On the x-axis, red indicates a positive phylogenetic autocorrelation and black indicates no phylogenetic autocorrelation.**

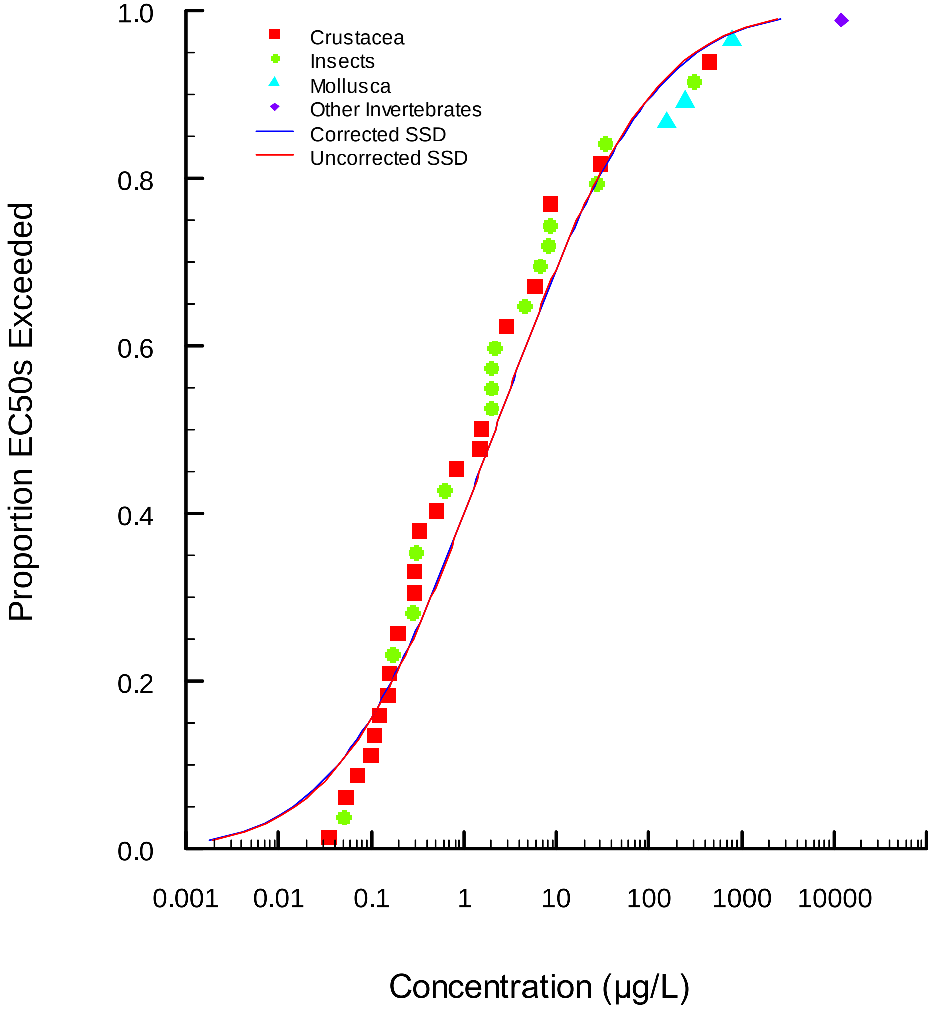

Figure SI-15 SSDs for the chlorpyrifos all aquatic invertebrates dataset showing original and corrected model fits.

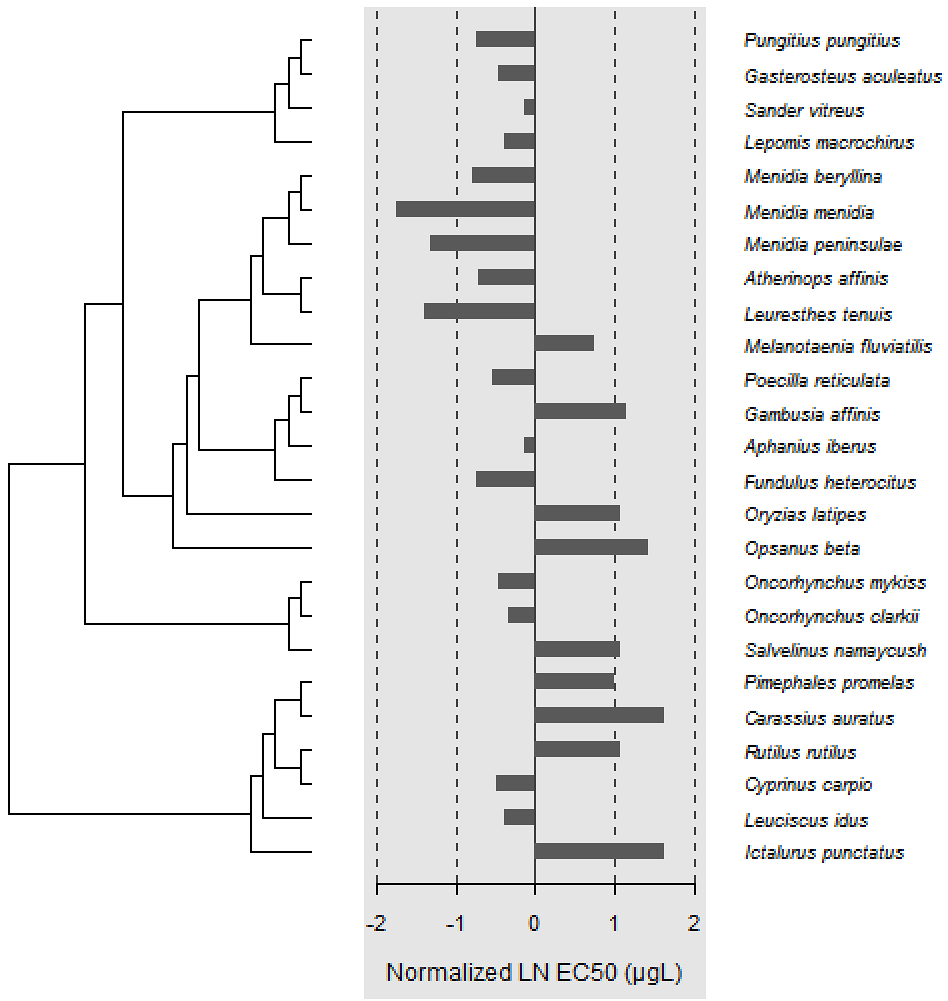

Figure SI-16 Bar plot showing normalized LN EC50 (i.e., median LN EC50=0) with respect to phylogenetic tree for the chlorpyrifos fish dataset.

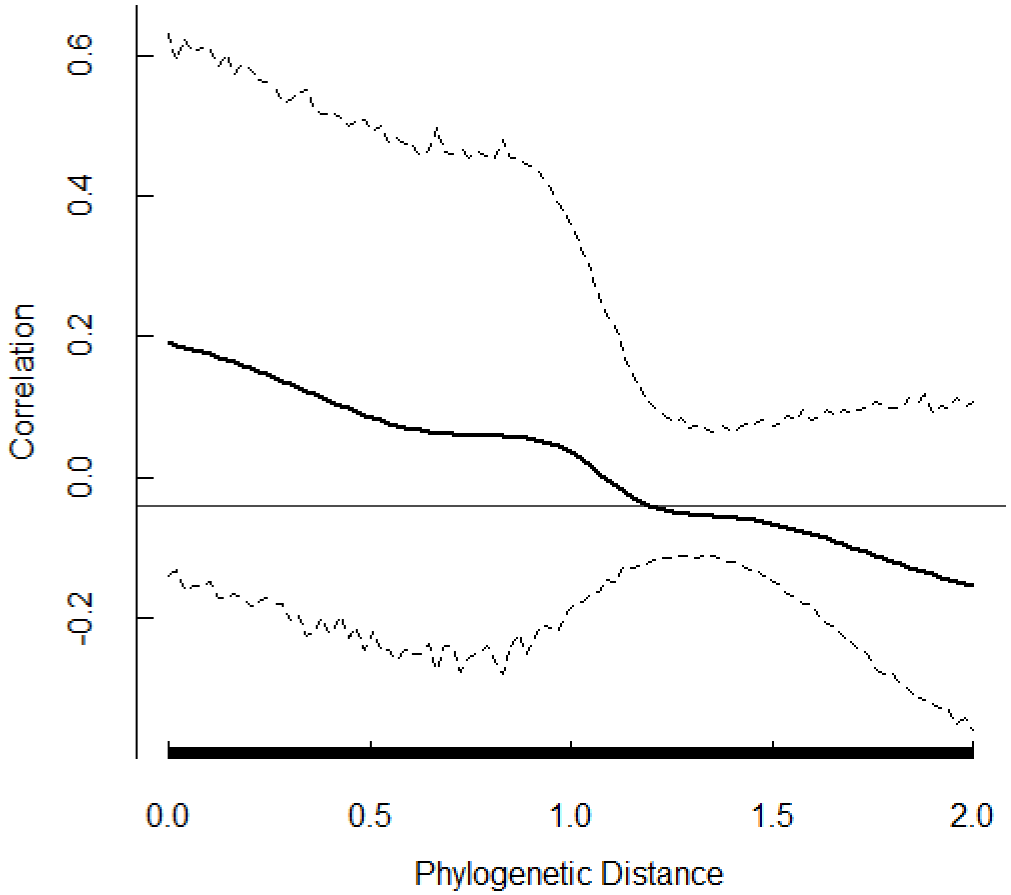

Figure SI-17 Correlogram for the chlorpyrifos fish dataset. On the x-axis, red indicates a positive phylogenetic autocorrelation and black indicates no phylogenetic autocorrelation.

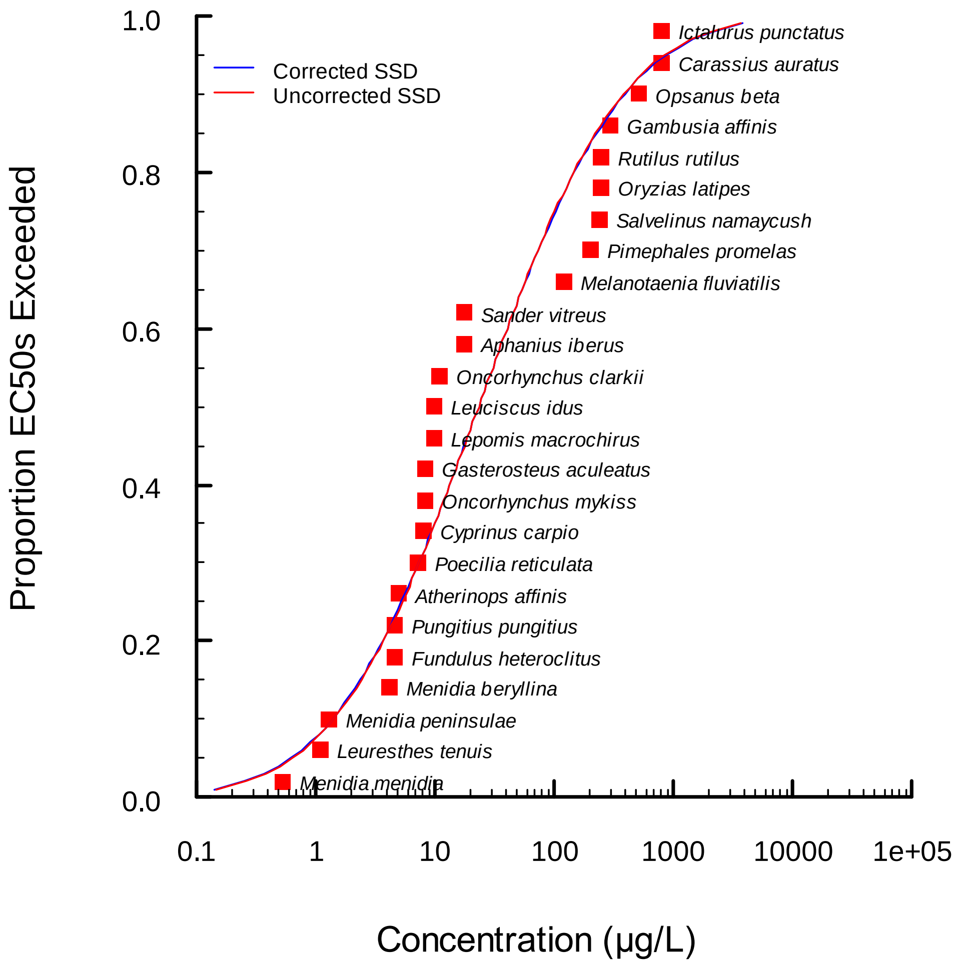

Figure SI-18 SSDs for the chlorpyrifos fish dataset showing original and corrected model fits.

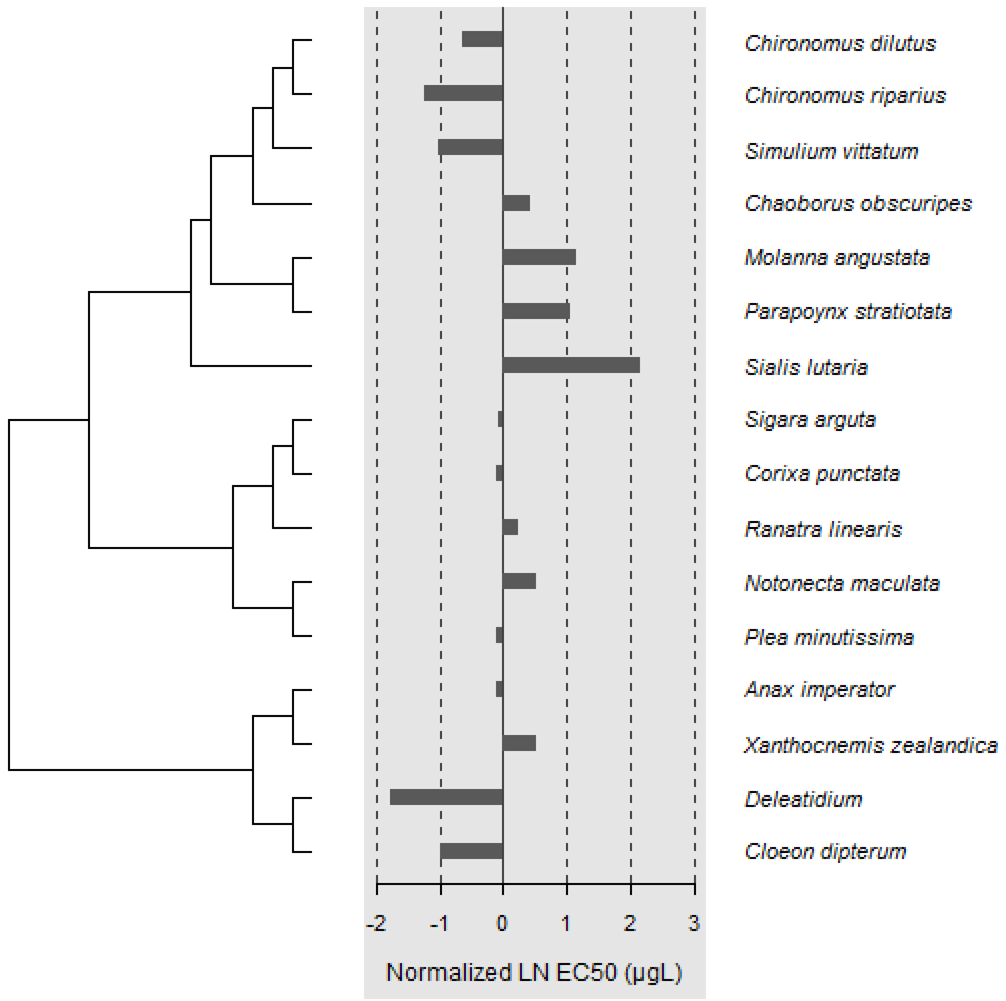

Figure SI-19 Bar plot showing normalized LN EC50 (i.e., median LN EC50=0) with respect to phylogenetic tree for the chlorpyrifos aquatic insects dataset.

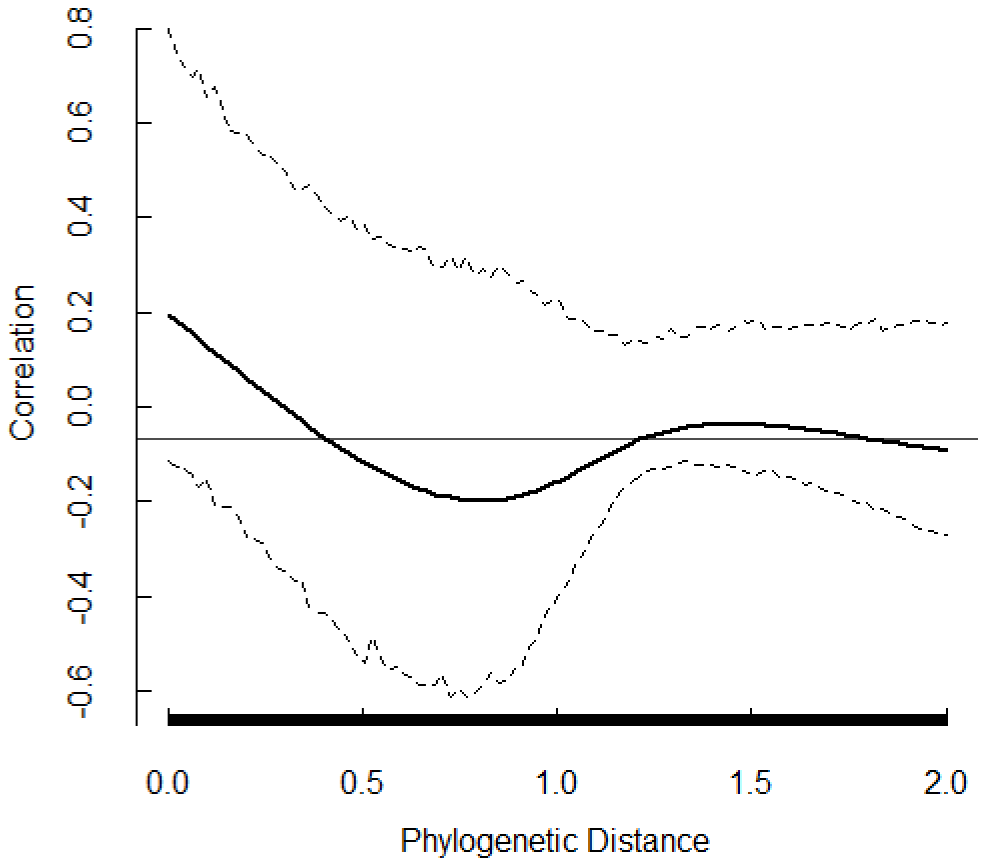

Figure SI-20 Correlogram for the chlorpyrifos aquatic insects dataset. On the x-axis, red indicates a positive phylogenetic autocorrelation and black indicates no phylogenetic autocorrelation.

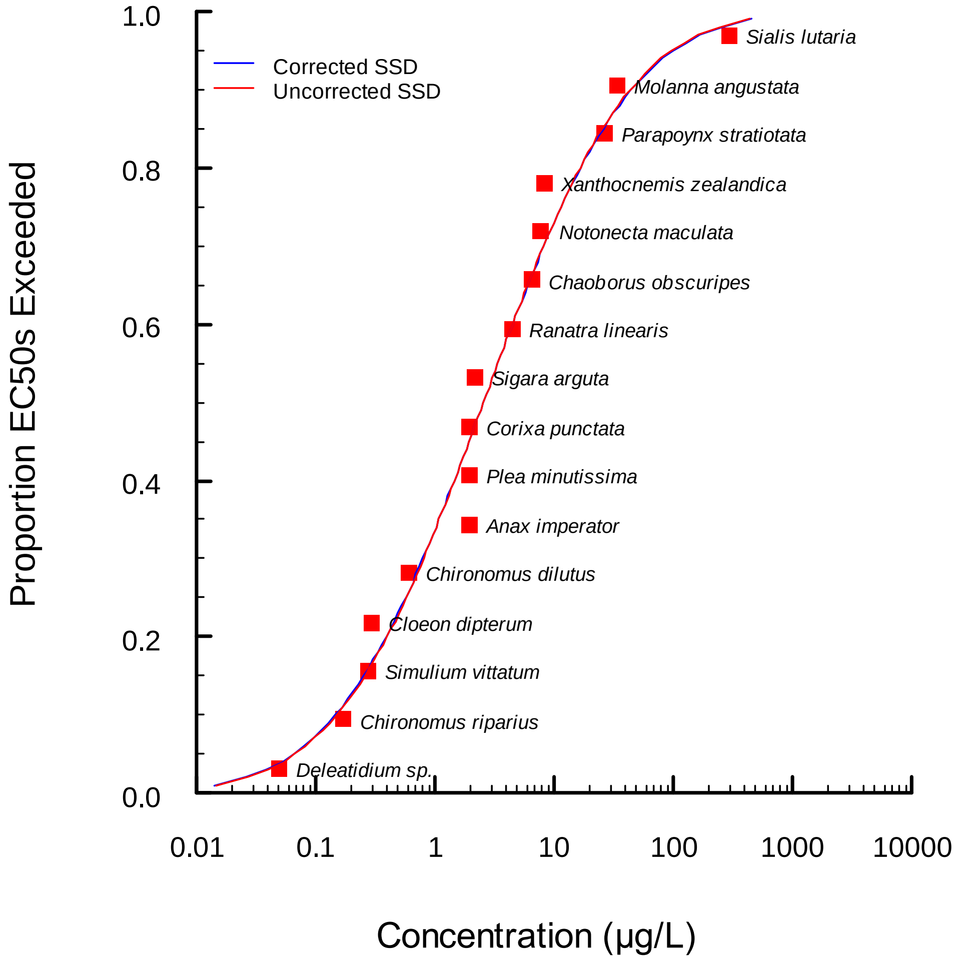

Figure SI-21 SSDs for the chlorpyrifos aquatic insects dataset showing original and corrected model fits.

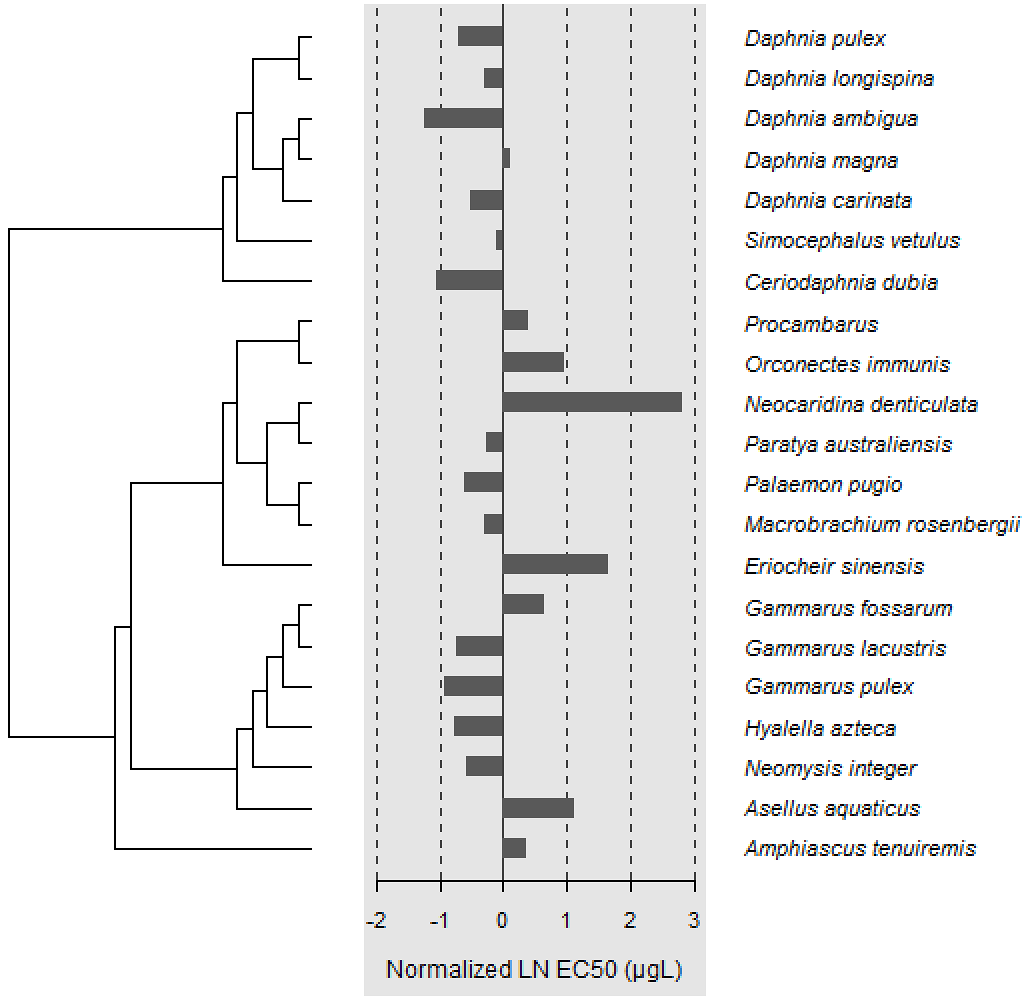

Figure SI-22 Bar plot showing normalized LN EC50 (i.e., median LN EC50=0) with respect to phylogenetic tree for chlorpyrifos crustaceans dataset.

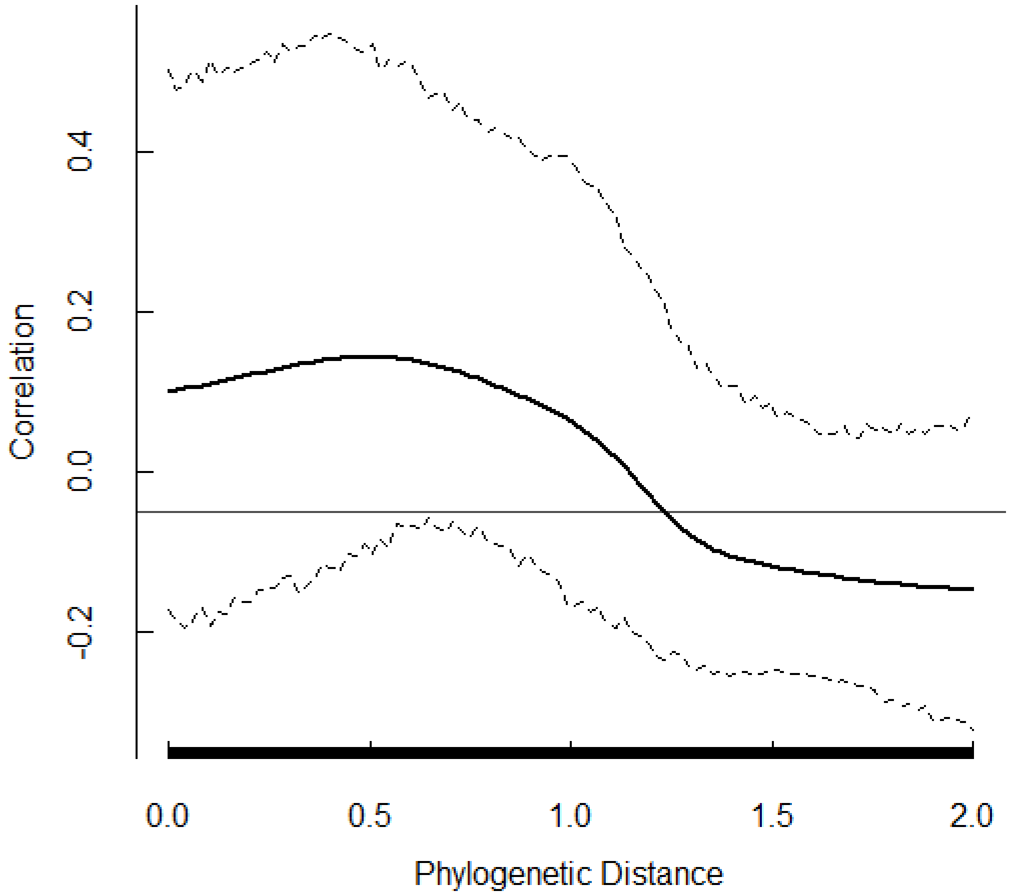

Figure SI-23 Correlogram for chlorpyrifos crustaceans dataset. On the x-axis, red indicates a positive phylogenetic autocorrelation and black indicates no phylogenetic autocorrelation.

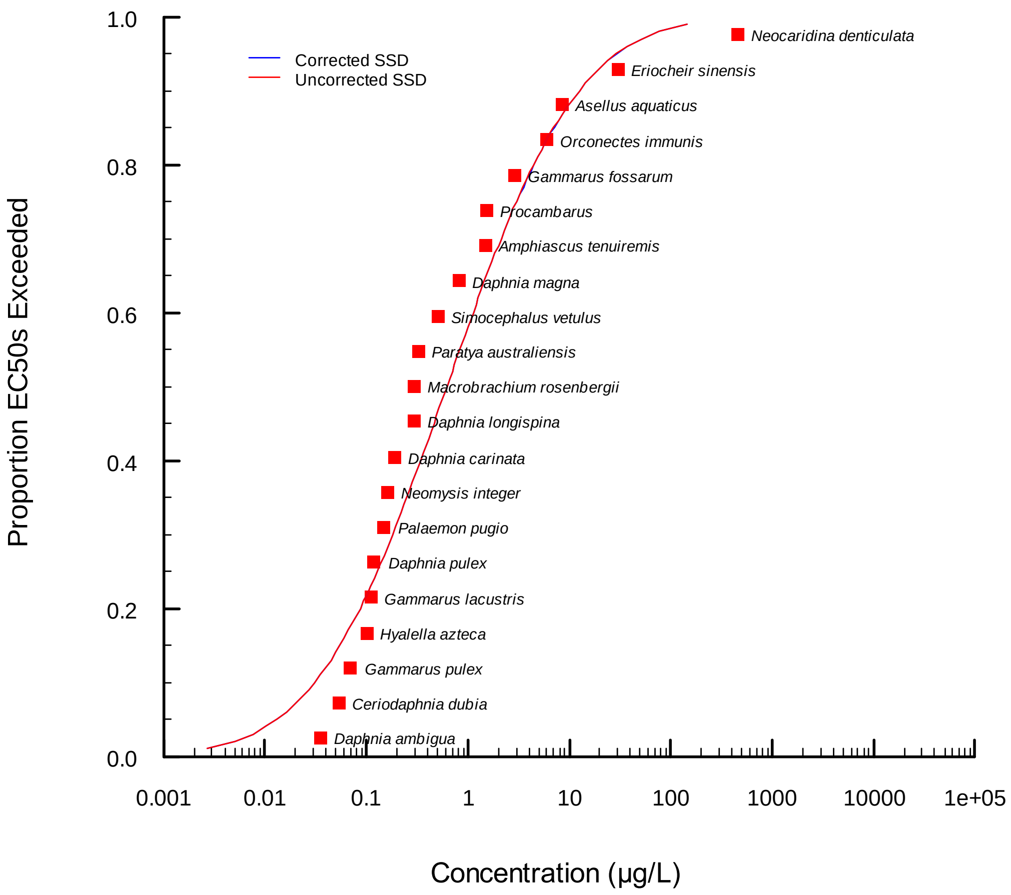

Figure SI-24 SSDs for chlorpyrifos crustaceans dataset showing original and corrected model fit.

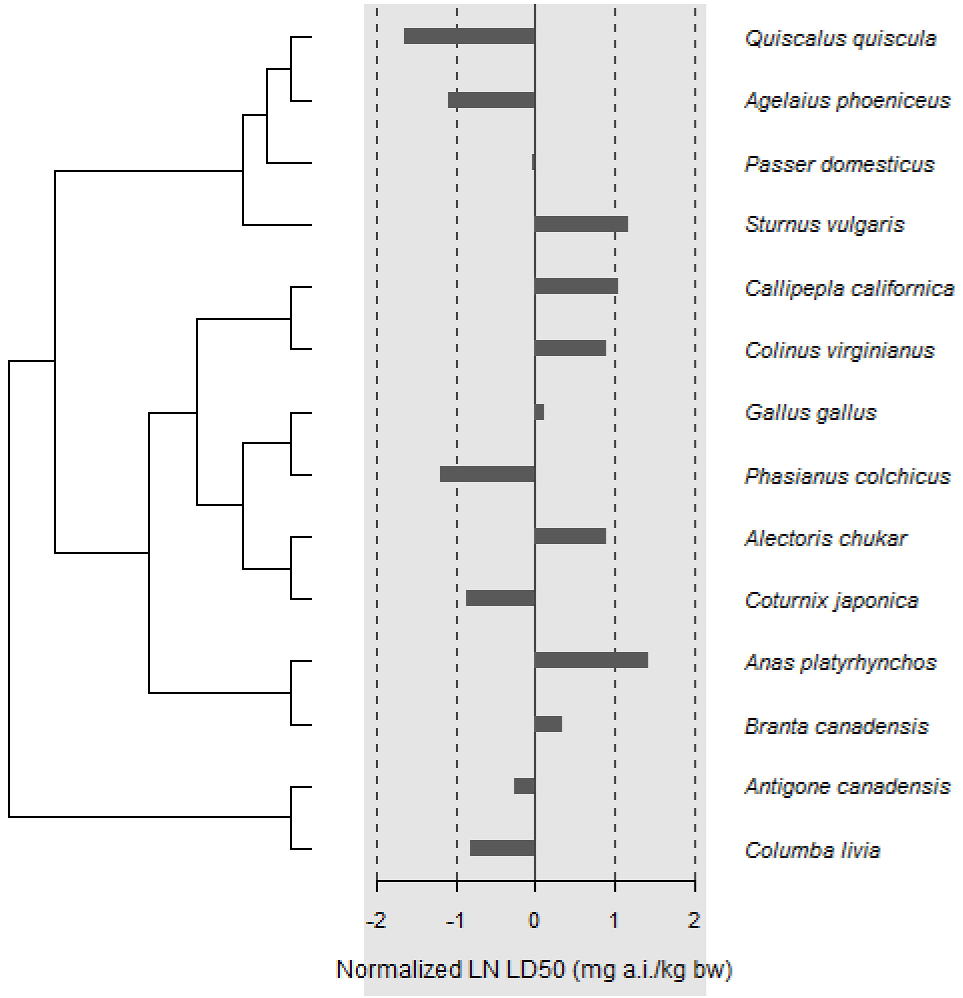

Figure SI-25 Bar plot showing normalized LN EC50 (i.e., median LN EC50=0) with respect to phylogenetic tree for chlorpyrifos birds dataset.

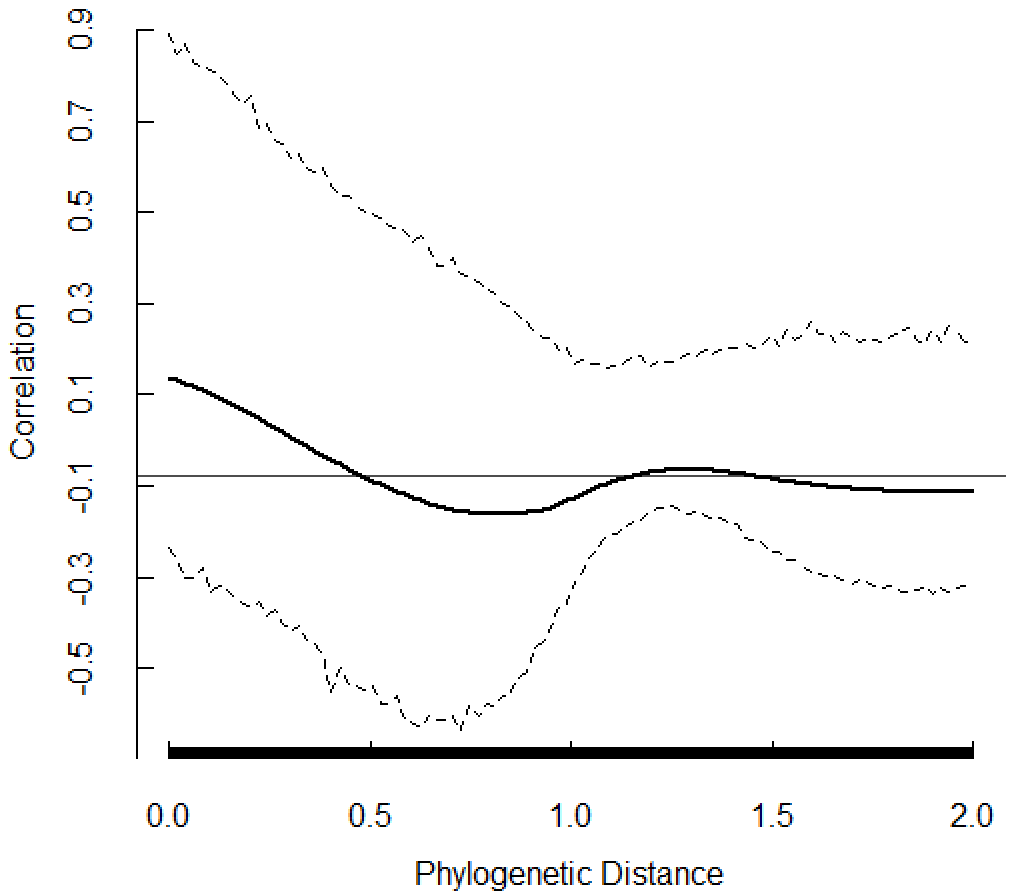

Figure SI-26 Correlogram for chlorpyrifos birds dataset. On the x-axis, red indicates a positive phylogenetic autocorrelation and black indicates no phylogenetic autocorrelation.

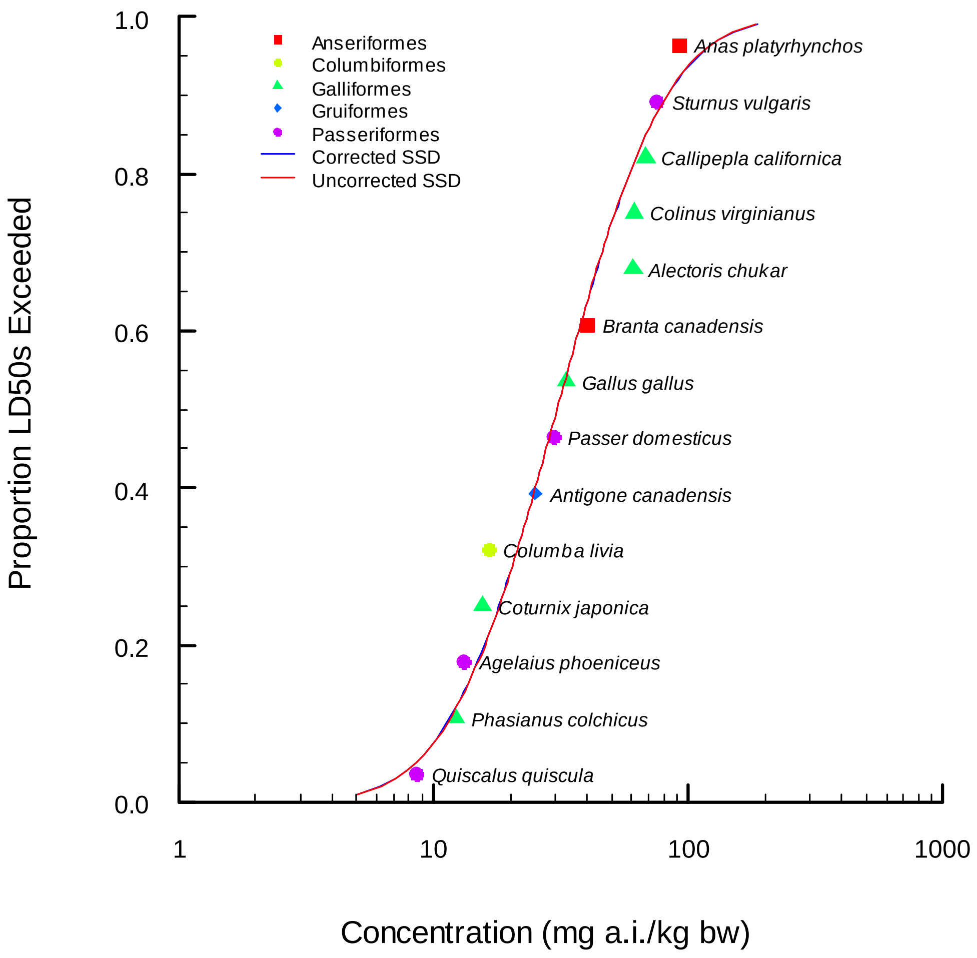

Figure SI-27 SSDs for chlorpyrifos birds dataset showing original and corrected model fit.

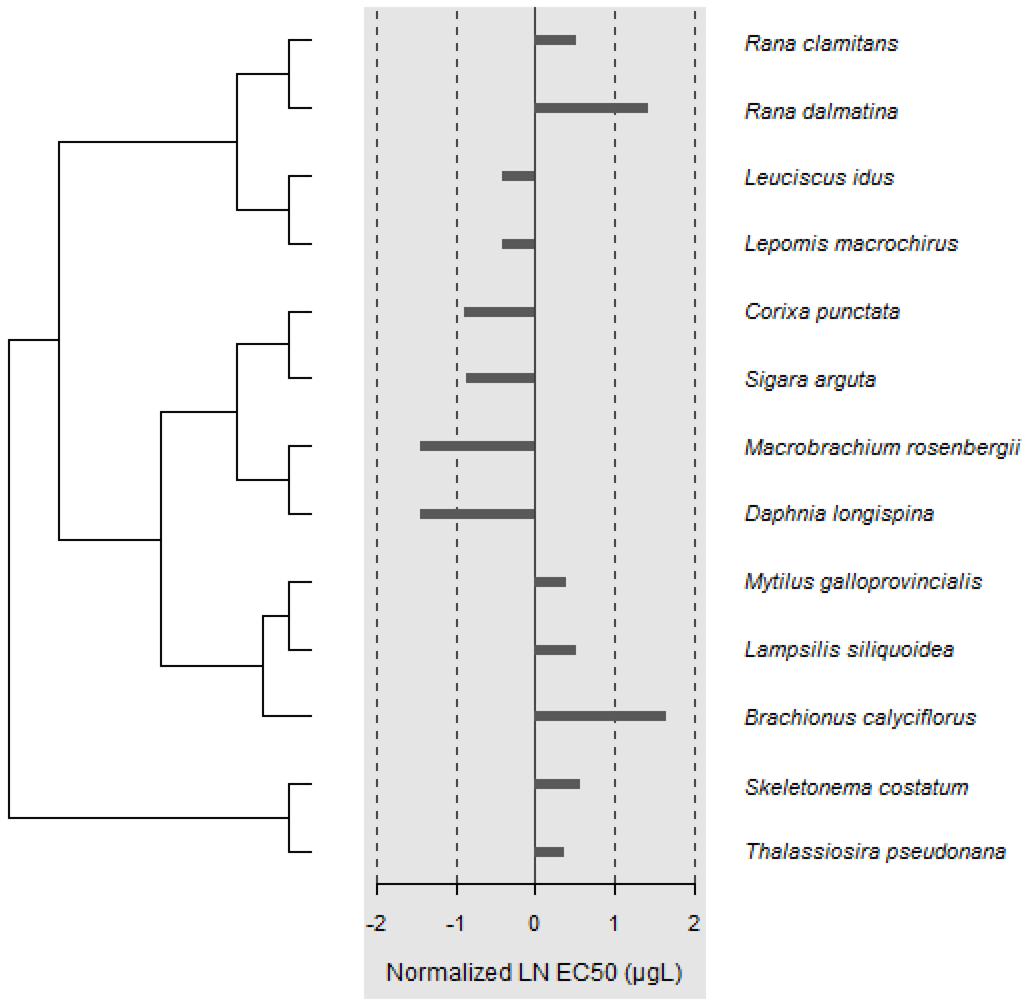

Figure SI-28 Bar plot showing normalized LN EC50 (i.e., median LN EC50=0) with respect to phylogenetic tree for first hypothetical chlorpyrifos aquatic dataset with a reduced sample size.

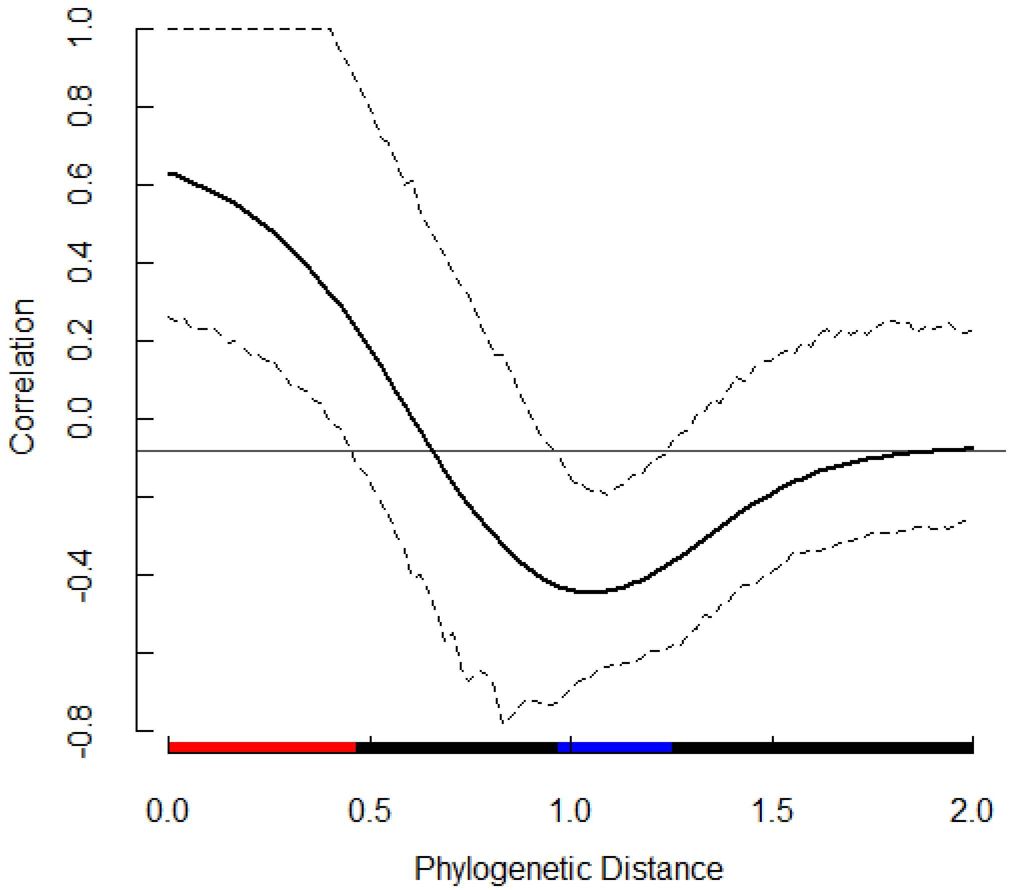

Figure SI-29 Correlogram for first hypothetical chlorpyrifos aquatic dataset. On the x-axis, red indicates a positive phylogenetic autocorrelation, blue a negative phylogenetic autocorrelation, and black indicates no phylogenetic autocorrelation.

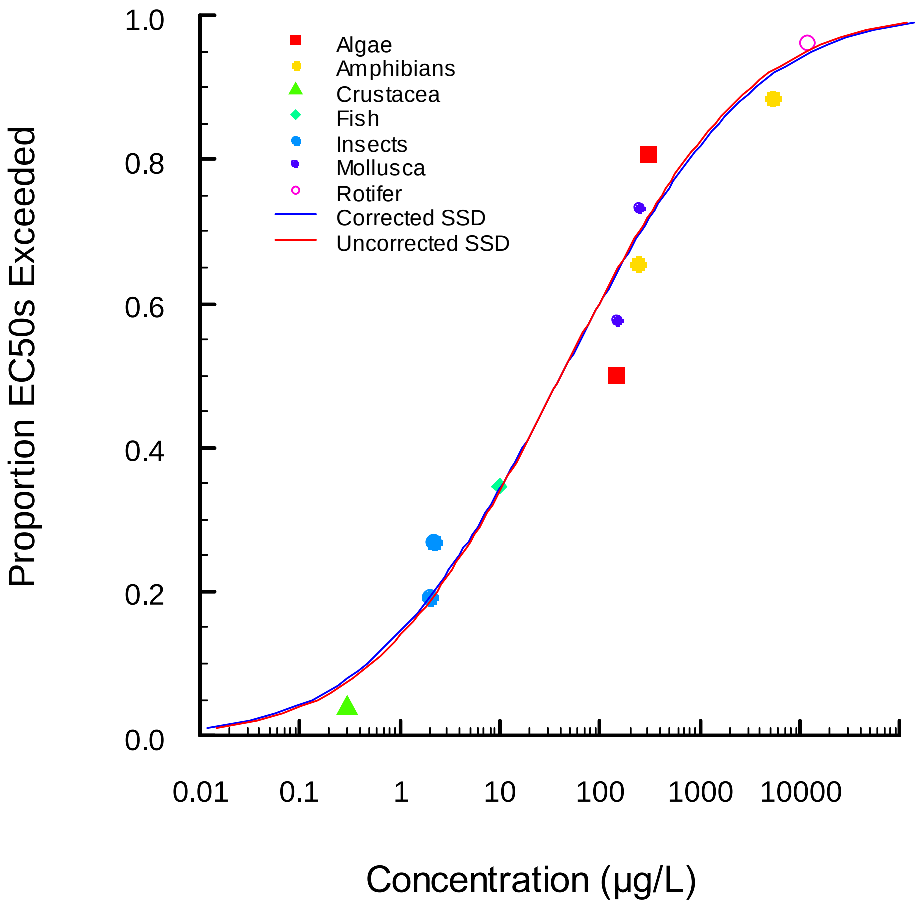

Figure SI-30 SSDs for first hypothetical chlorpyrifos aquatic dataset showing original and corrected model fit.

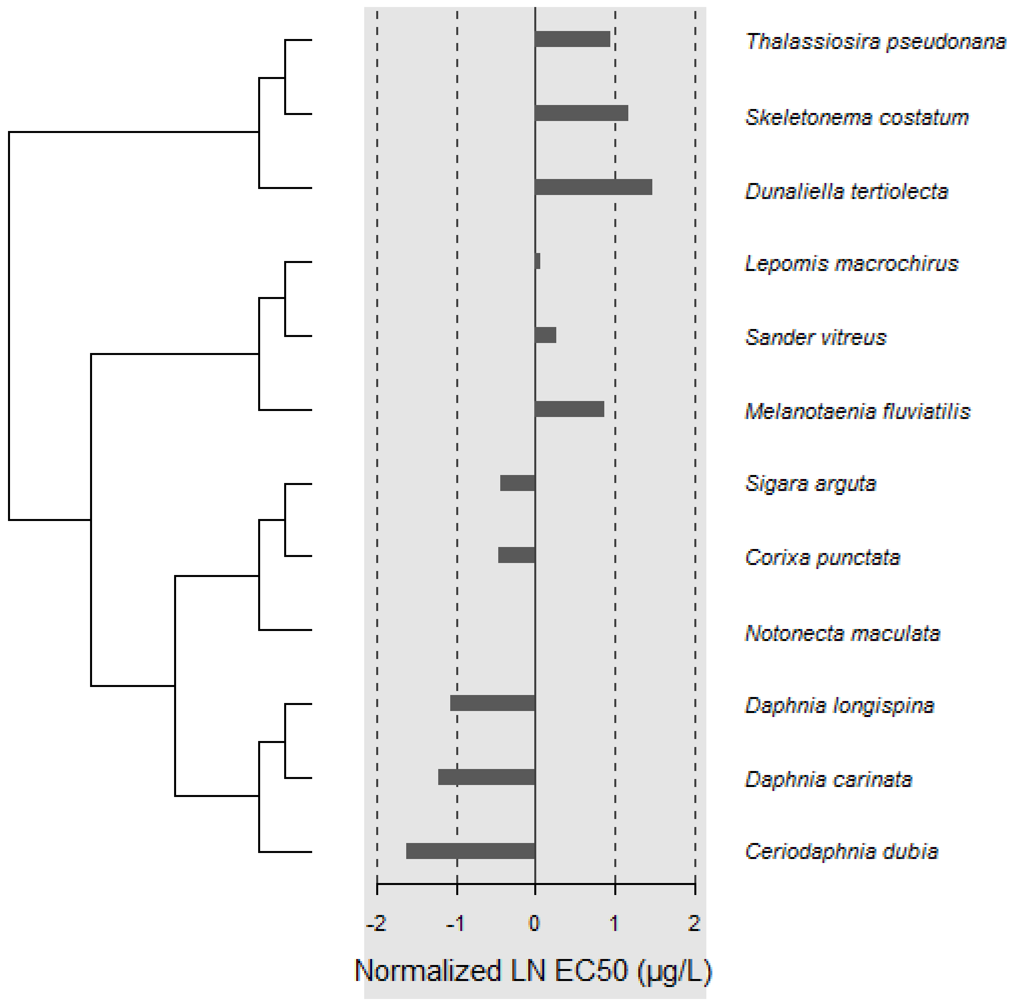

Figure SI-31 **Bar plot showing normalized LN EC50 (i.e., median LN EC50=0) with respect to phylogenetic tree for second hypothetical chlorpyrifos aquatic dataset with a reduced sample size.**

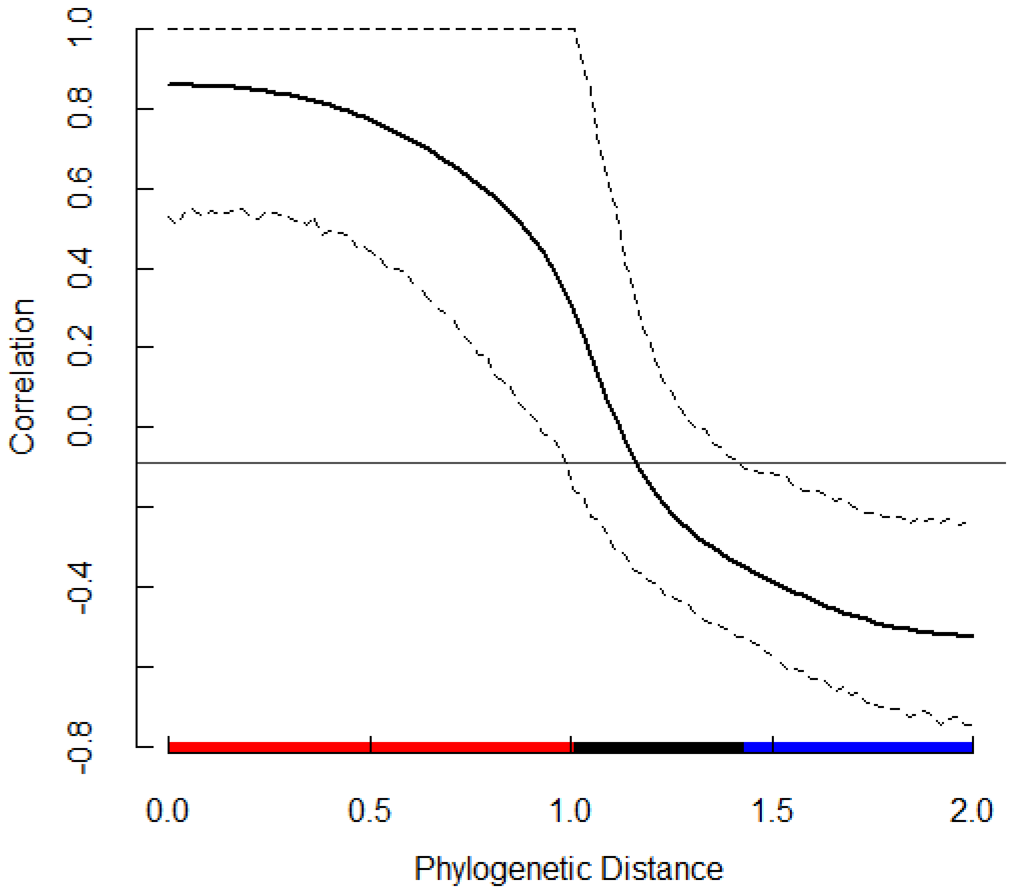

Figure SI-32 Correlogram for second hypothetical chlorpyrifos aquatic dataset. On the x-axis, red indicates a positive phylogenetic autocorrelation, blue indicates a negative phylogenetic autocorrelation, and black indicates no phylogenetic autocorrelation.

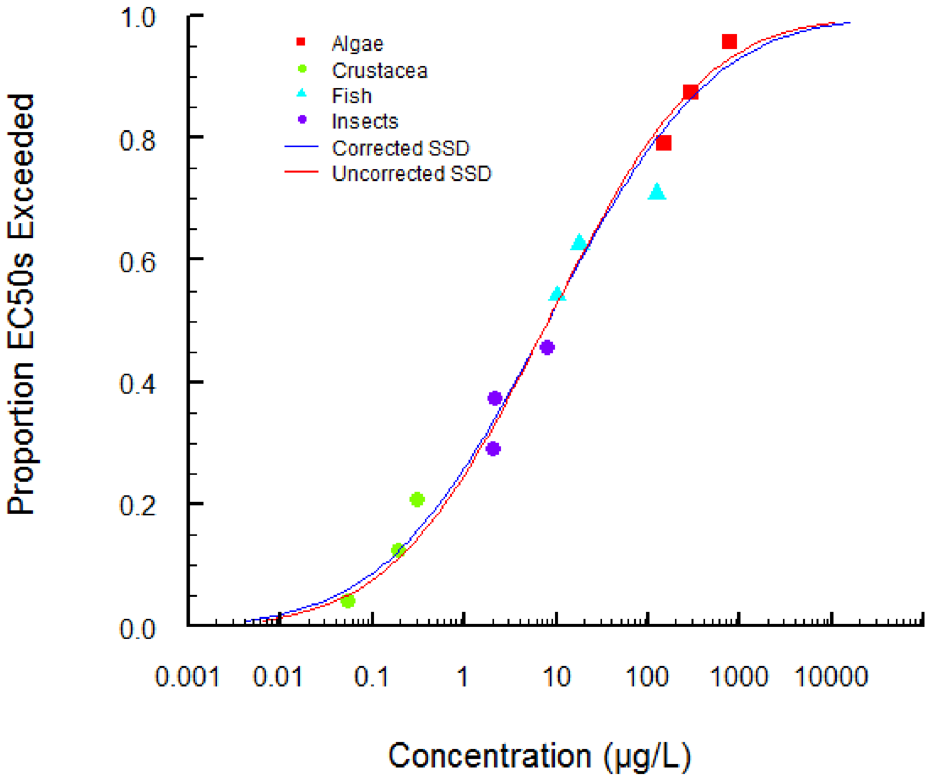

Figure SI-33 SSDs for second hypothetical chlorpyrifos aquatic dataset showing original and corrected model fit.

Tables

Table SI-1 Input data for atrazine aquatic plants dataset (USEPA 2012)

Taxon	***Species Name***	***NCBI Species Name***	***Taxonomic ID***	***SGR EC50 (µg/L)***	***Geomean Species SGR EC50 (µg/L)***
Cyanobacteria	*Anabaena cylindrica*	*Anabaena cylindrica*	1165	470	367
				286	
	Anabaena flos-aquae	*Dolichospermum flos-aquae*	1166	706	706
	Anabaena inaequalis	*Anabaena inaequalis*	445011	280	280
	Anabaena variabilis	*Trichormus variabilis*	264691	70	70
Archaeplastida	*Ankistrodesmus braunii*	*Chlorolobion braunii*	34112	104	104
	Ankistrodesmus sp.	*Ankistrodesmus*	3108	119	119
Angiospermae	*Ceratophyllum demersum*	*Ceratophyllum demersum*	4428	24	24
Archaeplastida	*Chlamydomonas noctigama*	*Chlamydomonas noctigama*	28456	378	378
	Chlamydomonas reinhardtii	*Chlamydomonas reinhardtii*	3055	141	75.2
				67	
				45	
	Chlorella fusca	*Desmodesmus abundans*	77547	26	26
	Chlorella pyrenoidosa	*Auxenochlorella pyrenoidosa*	3078	480	480
	Chlorella sp.	*Chlorella sp*	3079	37	37
	Chlorella vulgaris	*Chlorella vulgaris*	3077	91	225
				557	
Chromalveolata	*Cryptomonas pyrenoidifera*	*Cryptomonas pyrenoidifera*	233184	494	494
	Cyclotella meneghiana	*Cyclotella meneghiana*	29205	100	137
				114	
				225	
	Cyclotella sp.	*Cyclotella sp*	35126	462	462
Angiospermae	*Elodea canadensis*	*Elodea canadensis*	100364	65	79.6
				38	

Table SI-1 Input data for atrazine aquatic plants dataset (USEPA 2012)

Taxon	*Species Name*	*NCBI Species Name*	*Taxonomic ID*	*SGR EC50 (µg/L)*	*Geomean Species SGR EC50 (µg/L)*
				204	
Angiospermae	*Hydrilla verticillata*	*Hydrilla verticillata*	51024	118	118
	Lemna gibba	*Lemna gibba*	4470	202	113
				93	
				49	
				224	
				90	
	Lemna minor	*Lemna minor*	4472	115	105
				95	
Cyanobacteria	*Microcystis aeruginosa*	*Microcystis aeruginosa*	1126	164	315
				605	
Angiospermae	*Myriophyllum heterophyllum*	*Myriophyllum heterophyllum*	208866	150	150
	Najas sp.	*Najas*	13165	15	15
Chromalveolata	*Navicula pellculosa*	*Fistulifera pelliculosa*	913975	217	217
Angiospermae	*Potamogeton perfoliatus*	*Potamogeton perfoliatus*	55320	63	63
Archaeplastida	*Scenedesmus obliquus*	*Tetradesmus obliquus*	3088	87	87
	Scenedesmus quadricauda	*Scenedesmus quadricauda*	3089	300	300
	Scenedesmus subspicatus	*Desmodesmus subspicatus*	104105	39	39
	Selenastrum capricornutum	*Selenastrum capricornutum*	118073	164	123
				50	
				100	
				131	
				70	
				163	
				125	

Table SI-1 Input data for atrazine aquatic plants dataset (USEPA 2012)

Taxon	*Species Name*	*NCBI Species Name*	*Taxonomic ID*	*SGR EC50 (µg/L)*	*Geomean Species SGR EC50 (µg/L)*
				110	
				201	
				236	
				223	
				101	
				78	
	Stigeoclonium tenue	*Stigeoclonium tenue*	764117	317	317
Cyanobacteria	*Synechococcus leopoliensis*	*Synechococcus leopoliensis*	32047	136	136
Archaeplastida	*Ulothrix subconstricta*	*Gloeotila*	240455	159	159
Angiospermae	*Vallisneria americana*	*Vallisneria americana*	29649	141	141

Table SI-2 Input data for chlorpyrifos aquatic species dataset (Giddings et al. 2014)

Taxon	*Species Name*	*NCBI Species Name*	*Taxonomic ID*	*Geomean Effect Concentration (µg/L)[a]*
Algae	*Dunaliella tertiolecta*	*Dunaliella tertiolecta*	3047	769
	Isochrysis galbana	*Isochrysis galbana*	37099	138
	Skeletonema costatum	*Skeletonema costatum*	2843	298
	Thalassiosira pseudonana	*Thalassiosira pseudonana*	35128	148
Amphibian	*Lithobates clamitans clamitans*	*Rana clamitans*	145282	236
	Rana dalmatina	*Rana dalmatina*	51331	5,174
	Xenopus laevis	*Xenopus laevis*	8355	134
Crustacean	*Amphiascus tenuiremis*	*Amphiascus tenuiremis*	1042268	1.47
	Asellus aquaticus	*Asellus aquaticus*	92525	8.58
	Ceriodaphnia dubia	*Ceriodaphnia dubia*	117530	0.054
	Daphnia ambigua	*Daphnia ambigua*	77756	0.035
	Daphnia carinata	*Daphnia carinata*	120202	0.19

Table SI-2 Input data for chlorpyrifos aquatic species dataset (Giddings et al. 2014)

Taxon	***Species Name***	***NCBI Species Name***	***Taxonomic ID***	***Geomean Effect Concentration (μg/L)[a]***
	Daphnia longispina	*Daphnia longispina*	42846	0.3
	Daphnia magna	*Daphnia magna*	35525	0.82
	Daphnia pulex	*Daphnia pulex*	6669	0.12
	Eriocheir sinensis	*Eriocheir sinensis*	95602	30.5
	Gammarus fossarum	*Gammarus fossarum*	52638	2.9
	Gammarus lacustris	*Gammarus lacustris*	52639	0.11
	Gammarus pulex	*Gammarus pulex*	52641	0.07
	Hyalella azteca	*Hyalella azteca*	294128	0.1
	Macrobrachium rosenbergii	*Macrobrachium rosenbergii*	79674	0.3
	Neocaridina denticulata	*Neocaridina denticulata*	274642	457
	Neomysis integer	*Neomysis integer*	223650	0.16
	Orconectes immunis	*Orconectes immunis*	306176	6
	Palaemonetes pugio	*Palaemon pugio*	221654	0.15
	Paratya australiensis	*Paratya australiensis*	159741	0.33
	Procambarus sp	*Procambarus*	6726	1.55
	Simocephalus vetulus	*Simocephalus vetulus*	77651	0.5
Fish	*Aphanius iberus*	*Aphanius iberus*	136835	18
	Atherinops affinis	*Atherinops affinis*	238741	4.97
	Carassius auratus	*Carassius auratus*	7957	806
	Cyprinus carpio	*Cyprinus carpio*	7962	8
	Fundulus heteroclitus	*Fundulus heteroclitus*	8078	4.65
	Gambusia affinis	*Gambusia affinis*	33528	298
	Gasterosteus aculeatus	*Gasterosteus aculeatus*	69293	8.5
	Ictalurus punctatus	*Ictalurus punctatus*	7998	806
	Lepomis macrochirus	*Lepomis macrochirus*	13106	10
	Leuciscus idus	*Leuciscus idus*	69811	10
	Leuresthes tenuis	*Leuresthes tenuis*	355514	1.1

Table SI-2 Input data for chlorpyrifos aquatic species dataset (Giddings et al. 2014)

Taxon	*Species Name*	*NCBI Species Name*	*Taxonomic ID*	*Geomean Effect Concentration (µg/L)*[a]
	Melanotaenia fluviatilis	*Melanotaenia fluviatilis*	120844	122
	Menidia beryllina	*Menidia beryllina*	269057	4.2
	Menidia menidia	*Menidia menidia*	238744	0.53
	Menidia peninsulae	*Menidia peninsulae*	461517	1.3
	Oncorhynchus clarki	*Oncorhynchus clarkii*	30962	11
	Oncorhynchus mykiss	*Oncorhynchus mykiss*	8022	8.49
	Opsanus beta	*Opsanus beta*	95145	520
	Oryzias latipes	*Oryzias latipes*	8090	250
	Pimephales promelas	*Pimephales promelas*	90988	207
	Poecilia reticulata	*Poecilia reticulata*	8081	7.2
	Pungitius pungitius	*Pungitius pungitius*	134920	4.7
	Rutilus rutilus	*Rutilus rutilus*	48668	250
	Salvelinus namaycush	*Salvelinus namaycush*	8040	244
	Sander vitreus	*Sander vitreus*	283036	18
Insect	*Anax imperator*	*Anax imperator*	39274	1.98
	Chaoborus obscuripes	*Chaoborus obscuripes*	204562	6.6
	Chironomus dilutus	*Chironomus dilutus*	109233	0.62
	Chironomus riparius	*Chironomus riparius*	315576	0.17
	Cloeon dipterum	*Cloeon dipterum*	197152	0.3
	Corixa punctata	*Corixa punctata*	1545103	2
	Deleatidium sp.	*Deleatidium sp.*	551873	0.05
	Molanna angustata	*Molanna angustata*	446450	34
	Notonecta maculata	*Notonecta maculata*	1545171	7.97
	Parapoynx stratiotata	*Parapoynx stratiotata*	1594321	27.2
	Plea minutissima	*Plea minutissima*	280219	1.98
	Ranatra linearis	*Ranatra linearis*	280158	4.48
	Sialis lutariah	*Sialis lutaria*	279420	300

Table SI-2 Input data for chlorpyrifos aquatic species dataset (Giddings et al. 2014)

Taxon	***Species Name***	***NCBI Species Name***	***Taxonomic ID***	***Geomean Effect Concentration (μg/L)[a]***
	Sigara arguta	*Sigara arguta*	489481	2.16
	Simulium vittatum	*Simulium vittatum*	7192	0.28
	Xanthocnemis zealandica	*Xanthocnemis zealandica*	481685	8.44
Mollusc	*Aplexa hypnorum*	*Aplexa hypnorum*	271031	806
	Lampsilis siliquoidea	*Lampsilis siliquoidea*	52396	250
	Mytilus galloprovincialis	*Mytilus galloprovincialis*	29158	154
Rotifer	*Brachionus calyciflorus*	*Brachionus calyciflorus*	104777	12,000

[a] Greater than signs for *Carrasius auratus*, *Molanna angustata*, *Sialis lutiariah* and *Aplexa hypnorum* noted in Giddings et al. (2014) were dropped for our analysis

Table SI-3 Input data for chlorpyrifos birds dataset (Moore et al. 2014)

Order	***Species Name***	***NCBI Species Name***	***Taxonomic ID***	***Geomean Effect Concentration (μg/L)***
Anseriformes	*Anas platyrhynchos*	*Anas platyrhynchos*	8839	92
	Branta canadensis	*Branta canadensis*	8853	40
Columbiformes	*Columba livia*	*Columba livia*	8932	16.4
Galliformes	*Alectoris chukar*	*Alectoris chukar*	9078	60.9
	Callipepla californica	*Callipepla californica*	67771	68.3
	Colinus virginianus	*Colinus virginianus*	9014	61.7
	Coturnix japonica	*Coturnix japonica*	93934	15.6
	Gallus domesticus	*Gallus gallus*	9031	33.4
	Phasianus colchicus	*Phasianus colchicus*	9054	12.2
	Grus canadensis	*Antigone canadensis*	1977160	25
Passeriformes	*Agelaius phoeniceus*	*Agelaius phoeniceus*	39638	13.1
	Passer domesticus	*Passer domesticus*	48849	29.5
	Quiscalus quiscula	*Quiscalus quiscula*	84782	8.55
	Sturnus vulgaris	*Sturnus vulgaris*	9172	75

Example R Code (Chlorpyrifos Dataset for All Aquatic Species)

```
# Load required libraries
library(rJava)
library(maps)
library(dplyr)
library(ape)
library(phytools)
library(nlme)
library(xlsx)
library(Hmisc)
library(phylobase)
library(phylosignal)

# Import data from the SSD csv file
SSD_CPY_All <- read.csv(***insert name and hard drive location of csv file with NCBI species names, EC50 values, and Hazen
plotting positions***, header = TRUE, stringsAsFactors = FALSE, as.is = T)
attach(SSD_CPY_All)
dim(SSD_CPY_All) # Reports dimensions of SSD data set, i.e., numbers of columns and rows

# Define sample size
samplesize <-nrow(SSD_CPY_All)
samplesize

# Generate list of those species
species_list <- unique(SSD_CPY_All$NCBI_Species_Name)
species_list

# Add column of log transformed EC50 values to SSD_CPY_All
SSD_CPY_All$logEC50 <- log(EC50_µgL)
mu_hat <- mean(SSD_CPY_All$logEC50)
mu_hat
```

```
# Add a column to SSD_CPY_All containing (xi-xhat)^2. This column is needed later for variance estimation
SSD_CPY_All$SE <- (SSD_CPY_All$logEC50-mu_hat)^2
SSE <- sum(SSD_CPY_All$SE)
SSE

# Name of saved pdf file for all plots created below
pdf(***insert name and hard drive location for output plots files in pdf format***

# Plot imported data (Figure 1)
par(mfrow=c(1,1), family="sans", mai=c(0.3,0.3,0.3,0.3), omi=c(1,1,0.5,1))
plot(SSD_CPY_All$EC50_µgL,SSD_CPY_All$Hazen_Plotting_Position, xlim=c(0.01,100000), ylim=c(0,1), log="x", axes=FALSE,
cex=1.25, pch=c(15:21)[as.factor(SSD_CPY_All$Taxon)], col=rainbow(7)[as.factor(SSD_CPY_All$Taxon)])
axis(side=1, at=c(0.01,0.1,1,10,100,1000,10000,100000), pos=0, lwd=2, cex.axis=1, font=1, tck=0.02,
labels=c(0.01,0.1,1,10,100,1000,10000,100000))
axis(side=1, at=seq(0.01,0.1,0.01), pos=0, las=0, tck=0.01, labels=FALSE)
axis(side=1, at=seq(0.1,1,0.1), pos=0, las=0, tck=0.01, labels=FALSE)
axis(side=1, at=seq(1,10,1), pos=0, las=0, tck=0.01, labels=FALSE)
axis(side=1, at=seq(10,100,10), pos=0, las=0, tck=0.01, labels=FALSE)
axis(side=1, at=seq(100,1000,100), pos=0, las=0, tck=0.01, labels=FALSE)
axis(side=1, at=seq(1000,10000,1000), pos=0, las=0, tck=0.01, labels=FALSE)
axis(side=1, at=seq(10000,100000,10000), pos=0, las=0, tck=0.01, labels=FALSE)
axis(side=2, pos=0.01, lwd=2.5, cex.axis=1, font=1, las=1, tck=0.02)
axis(side=2, at=seq(0,1,0.05), pos=0.01, las=0, tck=0.01, labels=FALSE)
mtext("Concentration (µg/L)", side=1, outer=T, at=0.5, cex=1.25, line = 1.25)
mtext("Proportion EC50s Exceeded", side=2, outer=T, at=0.5, cex=1.25, line=1.25)
legend(x=0.1, y=1, legend=levels(factor(SSD_CPY_All$Taxon)), col=rainbow(7), cex=0.75, pch=c(15:21), bty="n")

# Import the newick file and save as a phy object to be used later for analysis and plotting
phy_fig <- read.newick(***insert name and hard drive location for newick text file in txt format***)

# Need to compute branch lengths on the tree, this is later needed for phylogenetic distances matrix
phy_figure <- compute.brlen(phy_fig, method="Grafen", power=1)
```

```
# Once computed, need to save as a phy object
write.tree(collapse.singles(phy_figure), "updated_Chlorpyrifos_all_aquatic_tree_fig.phy")

# Plot phylogenetic tree, cladogram (Figure 2)
tree_SSD <- read.tree("updated_Chlorpyrifos_all_aquatic_tree_fig.phy")
plot.phylo(tree_SSD, type="p", no.margin=T, edge.width=1, family="sans", font=3, cex=0.4, show.node.label=F, show.tip.label=T)
checkValidPhylo(tree_SSD)

# Obtain the phylogenetic distances matrix
tree.phydist <- cophenetic.phylo(tree_SSD)
write.xlsx(tree.phydist, ***insert name and hard drive location for output distances matrix Excel file in xlsx format***)

### IMPORTANT - Order of species in the toxicity dataset needs to be the same as in the phylogenetic tree
### This is done by re-arranging the order of species in the toxicity dataset to match the order in the phylogenetic tree
species_names <- c(tree_SSD$tip.label)
species_names
row.names(SSD_CPY_All) <- SSD_CPY_All$NCBI_Species_Name

# Make a phylo4d tree with logEC50s as trait for stats (Figure 3)
BaseTreeStats <- phylo4d(tree_SSD, SSD_CPY_All)
barplot(BaseTreeStats, trait="logEC50", bar.lwd=5, trait.labels="Normalized LN EC50 (µg/L)", trait.cex=0.75)

# Make a correlogram for trait=logEC50 (Figure 4)
logEC50.cg <- phyloCorrelogram(BaseTreeStats, trait="logEC50")
plot(logEC50.cg, main="", xlab="", ylab="", axes=FALSE)
axis(side=2, at=seq(-0.4,0.8,0.2), pos=0, tck=0.02)
axis(side=1, tck=0.02)
mtext("Phylogenetic Distance", side=1, line=2.5)
mtext("Correlation", side=2, line=2.5)

# Check for phylogenetic signal
stats <- phyloSignal(BaseTreeStats[,"logEC50"])
```

```
stats
write.xlsx(stats, ***insert name and hard drive location for output statistics Excel file in xlsx format***, sheetName="Stats",
row.names=FALSE)

# Retrieve Moran's I from results
statsdf <- data.frame(matrix(unlist(stats), nrow=5, byrow=T))
moran <- statsdf[1,2]
moran
write.xlsx(moran, ***insert name and hard drive location for output statistics Excel file in xlsx format***, sheetName="Moran",
row.names=FALSE, append=TRUE)

# Estimate effective sample size
Neff <- samplesize/(1+2*(moran/(1-moran))*(1-(1/samplesize))-2*(moran/(1-moran))^2*(((1-moran)^(samplesize-1))/samplesize))
Neff
write.xlsx(Neff, ***insert name and hard drive location for output statistics Excel file in xlsx format***, sheetName="Neff",
row.names=FALSE, append=TRUE)

# Estimate variance and standard deviation
s2_hat <- (Neff/((samplesize*(Neff-1))))*SSE
sd <- sqrt(s2_hat)
sd
write.xlsx(sd, ***insert name and hard drive location for output statistics Excel file in xlsx format***,
sheetName="Standard_Deviation", row.names=FALSE, append=TRUE)

# For comparison, calculate the uncorrected standard deviation
uncorrsd <- sd(SSD_CPY_All$logEC50)
uncorrsd
write.xlsx(uncorrsd, ***insert name and hard drive location for output statistics Excel file in xlsx format***,
sheetName="Uncorrected_Standard_Deviation", row.names=FALSE, append=TRUE)

# Determine lognormal SSD
p <- seq(0.01, 1, 0.01)
SSD <- data.frame(p)
```

```
library(stats)
SSD$x <- qlnorm(SSD$p, meanlog=mu_hat, sdlog=sd, lower.tail=TRUE, log.p=FALSE)
head(SSD)
write.xlsx(SSD, ***insert name and hard drive location for output statistics Excel file in xlsx format***, sheetName="SSD",
row.names=FALSE, append=TRUE)

# Plot the corrected SSD with the data (Figure 5)
par(mfrow=c(1,1), tcl=-0.4, family="sans", mai=c(0.3,0.5,0.5,0.3), omi=c(1,1,0.5,1))
plot(SSD_CPY_All $EC50_µgL,SSD_CPY_All $Hazen_Plotting_Position, xlim=c(0.01,100000), log="x", ylim=c(0,1),
axes=FALSE, las=1, pch=c(15:21)[as.factor(SSD_CPY_All $Taxon)], col=rainbow(7)[as.factor(SSD_CPY_All $Taxon)], cex=1.25)
lines(SSD$x, SSD$p)
axis(side=1, at=c(0.01,0.1,1,10,100,1000,10000,100000), pos=0, lwd=2, cex.axis=1, font=1, tck=0.02,
labels=c(0.01,0.1,1,10,100,1000,10000,100000))
axis(side=1, at=seq(0.01,0.1,0.01), pos=0, las=0, tck=0.01, labels=FALSE)
axis(side=1, at=seq(0.1,1,0.1), pos=0, las=0, tck=0.01, labels=FALSE)
axis(side=1, at=seq(1,10,1), pos=0, las=0, tck=0.01, labels=FALSE)
axis(side=1, at=seq(10,100,10), pos=0, las=0, tck=0.01, labels=FALSE)
axis(side=1, at=seq(100,1000,100), pos=0, las=0, tck=0.01, labels=FALSE)
axis(side=1, at=seq(1000,10000,1000), pos=0, las=0, tck=0.01, labels=FALSE)
axis(side=1, at=seq(10000,100000,10000), pos=0, las=0, tck=0.01, labels=FALSE)
axis(side=2, pos=0.01, lwd=2.5, cex.axis=1, font=1, las=1, tck=0.02)
axis(side=2, at=seq(0,1,0.05), pos=0.01, las=0, tck=0.01, labels=FALSE)
mtext("Concentration (µg/L)", side=1, outer=T, at=0.5, cex=1.25, line=1.25)
mtext("Proportion EC50s Exceeded", side=2, outer=T, at=0.5, cex=1.25, line=1.25)
legend(x=0.05, y=1, legend=c(levels(factor(SSD_CPY_All $Taxon)), "SSD"), col=c(rainbow(7), "black"), cex=0.75, pch=c(15:21,
NA), lty=c(NA,NA,NA,NA,NA,NA,NA,1), bty="n")

# For comparison, determine the uncorrected lognormal SSD
nonp <- seq(0.01, 1, 0.01)
uncorrSSD <- data.frame(nonp)
uncorrSSD$x <- qlnorm(uncorrSSD$nonp, meanlog=mu_hat, sdlog=sd(SSD_CPY_All $logEC50), lower.tail=TRUE, log.p=FALSE)
head(uncorrSSD)
```

```
write.xlsx(uncorrSSD, ***insert name and hard drive location for output statistics Excel file in xlsx format***,
sheetName="Uncorrected_SSD", row.names=FALSE, append=TRUE)

# Plot corrected and uncorrected lognormal SSDs together (Figure 6)
par(mfrow=c(1,1), tcl=-0.4, family="sans", mai=c(0.3,0.5,0.5,0.3), omi=c(1,1,0.5,1))
plot(SSD_CPY_All$EC50_µgL,SSD_CPY_All $Hazen_Plotting_Position, xlim=c(0.01,100000), ylim=c(0,1), log="x",
axes=FALSE, cex=1.25, pch=c(15:21)[as.factor(SSD_CPY_All$Taxon)], col=rainbow(7)[as.factor(SSD_CPY_All $Taxon)])
lines(SSD$x, SSD$p, col="blue")
lines(uncorrSSD$x, uncorrSSD$nonp, col="red")
axis(side=1, at=c(0.01,0.1,1,10,100,1000,10000,100000), pos=0, lwd=2, cex.axis=1, font=1, tck=0.02,
labels=c(0.01,0.1,1,10,100,1000,10000,100000))
axis(side=1, at=seq(0.01,0.1,0.01), pos=0, las=0, tck=0.01, labels=FALSE)
axis(side=1, at=seq(0.1,1,0.1), pos=0, las=0, tck=0.01, labels=FALSE)
axis(side=1, at=seq(1,10,1), pos=0, las=0, tck=0.01, labels=FALSE)
axis(side=1, at=seq(10,100,10), pos=0, las=0, tck=0.01, labels=FALSE)
axis(side=1, at=seq(100,1000,100), pos=0, las=0, tck=0.01, labels=FALSE)
axis(side=1, at=seq(1000,10000,1000), pos=0, las=0, tck=0.01, labels=FALSE)
axis(side=1, at=seq(10000,100000,10000), pos=0, las=0, tck=0.01, labels=FALSE)
axis(side=2, pos=0.01, lwd=2.5, cex.axis=1, font=1, las=1, tck=0.02)
axis(side=2, at=seq(0,1,0.05), pos=0.01, las=0, tck=0.01, labels=FALSE)
mtext("Concentration (µg/L)", side=1, outer=T, at=0.5, cex=1.25, line=1.25)
mtext("Proportion EC50s Exceeded", side=2, outer=T, at=0.5, cex=1.25, line=1.25)
legend(x=0.05, y=1, legend=c(levels(factor(SSD_CPY_All $Taxon)), "Corrected SSD", "Uncorrected SSD"), col=c(rainbow(7),
"blue", "red"), cex=0.75, pch=c(15:21, NA, NA), lty=c(NA,NA,NA,NA,NA,NA,NA,1,1), bty="n")

dev.off()
```
